# Phosphorylation of *Toxoplasma gondii* Secreted Proteins during Acute and Chronic Stages of Infection

**DOI:** 10.1128/mSphere.00792-20

**Published:** 2020-09-09

**Authors:** Joanna C. Young, Malgorzata Broncel, Helena Teague, Matt R. G. Russell, Olivia L. McGovern, Matt Renshaw, David Frith, Ambrosius P. Snijders, Lucy Collinson, Vern B. Carruthers, Sarah E. Ewald, Moritz Treeck

**Affiliations:** a Signalling in Apicomplexan Parasites Laboratory, The Francis Crick Institute, London, United Kingdom; b Electron Microscopy Science Technology Platform, The Francis Crick Institute, London, United Kingdom; c Department of Microbiology and Immunology, University of Michigan Medical School, Ann Arbor, Michigan, USA; d Advanced Light Microscopy Science Technology Platform, The Francis Crick Institute, London, United Kingdom; e Proteomics Science Technology Platform, The Francis Crick Institute, London, United Kingdom; f Department of Microbiology, Immunology and Cancer Biology, University of Virginia, Charlottesville, Virginia, USA; g Carter Immunology Center, University of Virginia, Charlottesville, Virginia, USA; University at Buffalo

**Keywords:** host-pathogen interaction, virulence factors, chronic infection, protein phosphorylation

## Abstract

The intracellular parasite Toxoplasma gondii resides within a membrane-bound parasitophorous vacuole (PV) and secretes an array of proteins to establish this replicative niche. It has been shown previously that *Toxoplasma* secretes kinases and that numerous proteins are phosphorylated after secretion. Here, we assess the role of the phosphorylation of strand-forming protein 1 (SFP1) and the related protein GRA29, two secreted proteins with unknown function. We show that both proteins form stranded structures in the PV that are independent of the previously described intravacuolar network or actin. SFP1 and GRA29 can each form these structures independently of other *Toxoplasma* secreted proteins, although GRA29 appears to regulate SFP1 strands. We show that an unstructured region at the C termini of SFP1 and GRA29 is required for the formation of strands and that mimicking the phosphorylation of this domain of SFP1 negatively regulates strand development. When tachyzoites convert to chronic-stage bradyzoites, both proteins show a dispersed localization throughout the cyst matrix. Many secreted proteins are reported to dynamically redistribute as the cyst forms, and secreted kinases are known to play a role in cyst formation. Using quantitative phosphoproteome and proteome analyses comparing tachyzoite and early bradyzoite stages, we reveal widespread differential phosphorylation of secreted proteins. While we found no direct evidence for phosphorylation playing a dominant role for SFP1/GRA29 redistribution in the cyst, these data support a model in which secreted kinases and phosphatases contribute to the regulation of secreted proteins during stage conversion.

**IMPORTANCE** Toxoplasma gondii is a common parasite that infects up to one-third of the human population. Initially, the parasite grows rapidly, infecting and destroying cells of the host, but subsequently switches to a slow-growing form and establishes chronic infection. In both stages, the parasite lives within a membrane-bound vacuole within the host cell, but in the chronic stage, a durable cyst wall is synthesized, which provides protection to the parasite during transmission to a new host. *Toxoplasma* secretes proteins into the vacuole to build its replicative niche, and previous studies identified many of these proteins as phosphorylated. We investigate two secreted proteins and show that a phosphorylated region plays an important role in their regulation in acute stages. We also observed widespread phosphorylation of secreted proteins when parasites convert from acute to chronic stages, providing new insight into how the cyst wall may be dynamically regulated.

## Abstract (precis)

Toxoplasma gondii is a common parasite that infects up to one-third of the human population. Initially, the parasite grows rapidly, infecting and destroying cells of the host, but subsequently switches to a slow-growing form and establishes chronic infection. In both stages, the parasite lives within a membrane-bound vacuole within the host cell, but in the chronic stage, a durable cyst wall is synthesized, which provides protection to the parasite during transmission to a new host. *Toxoplasma* secretes proteins into the vacuole to build its replicative niche, and previous studies identified many of these proteins as phosphorylated. We investigate two secreted proteins and show that a phosphorylated region plays an important role in their regulation in acute stages. We also observed widespread phosphorylation of secreted proteins when parasites convert from acute to chronic stages, providing new insight into how the cyst wall may be dynamically regulated.

## INTRODUCTION

The apicomplexan parasite Toxoplasma gondii is widespread, infecting approximately one-third of the world’s population (1). Although *Toxoplasma* infection is predominantly asymptomatic in healthy hosts, complications occur in the immunocompromised, such as cancer and HIV patients, and in pregnant women. In these patients, infection can cause encephalitis or severely damage the unborn fetus during pregnancy (2). Additionally, *Toxoplasma* is a leading cause of retinochoroiditis (3), and an expansion of strain diversity in South America is causing substantial ocular disease (4).

*Toxoplasma* actively invades nucleated cells of virtually any warm-blooded animal and subsequently replicates within a membrane-bound parasitophorous vacuole (PV). During the acute stage of infection, the rapidly growing tachyzoites complete rounds of invasion and lysis of host cells and disseminate throughout the body. Upon immune pressure or other stress, the parasite converts to slow-growing bradyzoites that form cysts, predominantly in skeletal muscle and the brain (5). The cyst is a modified PV with a protective cyst wall that allows the parasite to establish chronic, perhaps lifelong, infection of the host.

During infection, *Toxoplasma* secretes proteins that subvert host cell signaling and develops its replicative niche within the PV (6, 7). The repertoire of secreted proteins is thought to include up to 200 proteins (8), including 50 kinases/pseudokinases (9), although the functions of many of these remain unknown. These proteins are secreted from the rhoptries or dense granules and are called ROPs or GRAs, respectively. Secreted proteins extensively modify the PV, allowing the selective passage of molecules and proteins across the PV membrane. For example, GRA17 and GRA23 form pores in the PV membrane, allowing the passage of small molecules into the PV (10), while the MYR complex mediates the transport of secreted proteins to the host cell (11, 12). Additionally, the parasite develops a complex set of membrane structures within the PV, including GRA7-lined invaginations (13) and a network of tubules called the intravacuolar network (IVN) (14). The formation of this network is dependent on GRA2 and the accessory protein GRA6 and is required for full virulence in mice (15, 16). Both structures have been reported to play a role in scavenging nutrients from the host cell with the uptake of host cell proteins (17), lipid droplets (18), and endolysosomal compartments (19).

A previous phosphoproteome analysis revealed that a large number of *Toxoplasma* secreted proteins are phosphorylated after their release (20), raising the intriguing possibility that their function is dynamically regulated. Indeed, the PV-localized kinase WNG1 was shown to contribute to the formation of a functional IVN (21). Furthermore, it was recently shown that IVN-associated GRAs show dynamic localization patterns as the parasite remodels the PV to form the chronic-stage cyst (22). Here, we analyze two secreted, strand-forming proteins that are phosphorylated after secretion and show that their phosphorylated C-terminal tails regulate their localization, disrupting normal strand formation. Both proteins disperse in chronic-stage cysts, so we hypothesized a role for phosphorylation and expanded the analysis to compare the phosphoproteomes of acute- and chronic-stage parasites. While SFP1/GRA29 dispersion in the forming cyst appears not to be primarily regulated by phosphorylation, we show that many GRAs are differentially phosphorylated between these stages, suggesting that the phosphorylation of secreted proteins may be a key determinant for the dynamic restructuring of the replicative niche of *Toxoplasma*.

(This article was submitted to an online preprint archive [23].)

## RESULTS

### A secreted protein forms strand-like structures in the parasitophorous vacuole independently of the intravacuolar network and actin.

**FIG 1:**
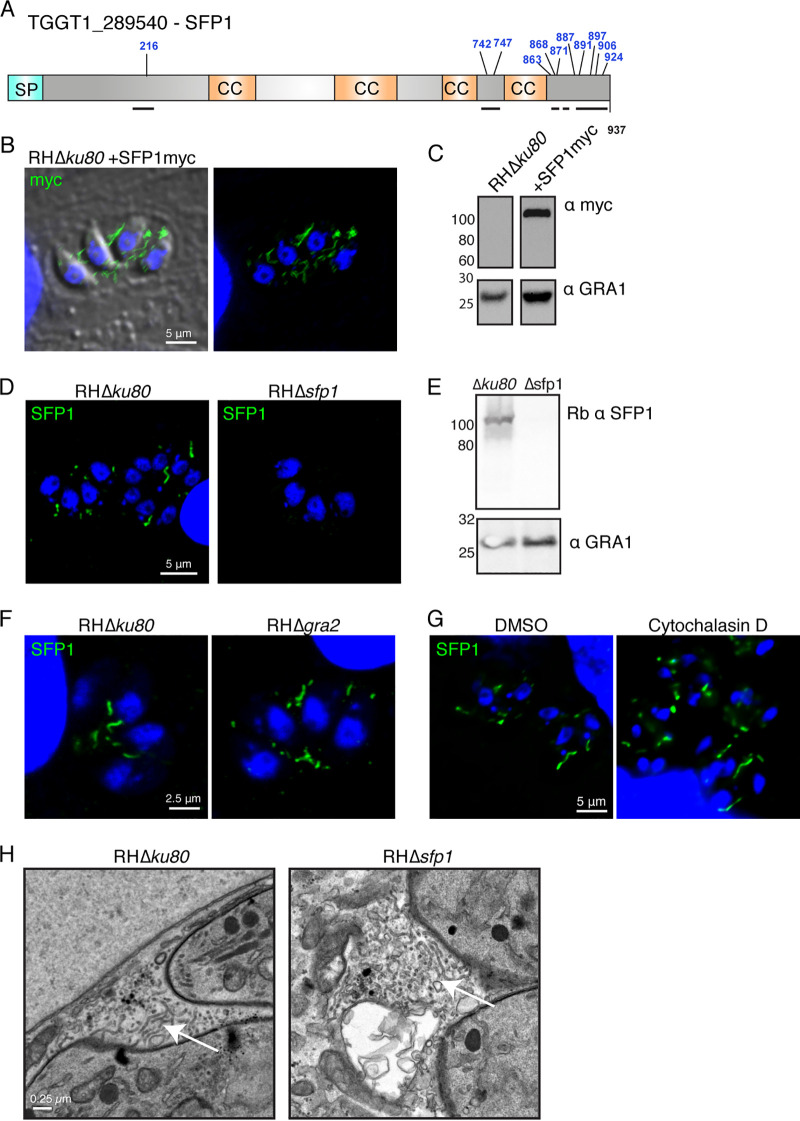
SFP1 forms strands in the parasitophorous vacuole that are distinct from the intravacuolar network. (A) Schematic of TGGT1_289540 (SFP1) indicating predicted domains and previously identified phosphosites (shown in blue). SP, signal peptide; CC, coiled coil. Black lines show predicted unstructured regions. (B) Immunofluorescence images of HFFs infected with RHΔ*ku80* parasites expressing SFP1-myc. Green, anti-myc antibody; blue, DAPI. (C) Western blot of immunoprecipitated TGGT1_289540::myc. Anti-GRA1 antibodies were used to probe the nonbound fraction as a loading control. (D) Immunofluorescence images of HFFs infected with RHΔ*ku80* and RHΔ*sfp1* parasites. Green, polyclonal rabbit anti-SFP1 antibody; blue, DAPI. (E) Western blot analysis of lysates from HFFs infected with RHΔ*ku80* and RHΔ*sfp1* parasites probed with rabbit (rb) anti-SFP1 and anti-GRA1 antibodies. (F) SFP1 strands do not depend on GRA2 and the IVN. Immunofluorescence images of RHΔ*ku80*- and RHΔ*gra2*-infected HFFs. Green, rabbit anti-SFP1 antibody; blue, DAPI. (G) SFP1 strands do not depend on actin filamentation. Immunofluorescence images of infected HFFs treated with 1 μM cytochalasin D or the DMSO control. Green, rabbit anti-SFP1 antibody; blue, DAPI. (H) SFP1 is not required for IVN formation. Electron micrographs of HFFs infected with RHΔ*ku80* and RHΔ*sfp1* for 16 h. Examples of intravacuolar network tubules are indicated by arrows.

To investigate the role of the phosphorylation of secreted proteins, we selected TGGT1_289540 as an ideal candidate. It is substantially phosphorylated after secretion (20) and contains a localized cluster of phosphorylation sites in its C terminus, allowing for targeted genetic mutagenesis. TGGT1_289540 contains a signal peptide and is predicted to localize to the dense granules (8). It has four predicted coiled-coil domains and an unstructured C terminus that contains 8 phosphorylation sites (Fig. 1A). Three further phosphorylation sites are located within the rest of the protein. To first verify that TGGT1_289540 is a secreted protein, we expressed a myc-tagged version of the protein, which localized to the PV. Interestingly, in contrast to most GRAs that either are found to fill the space between parasites or are found associated with the PV membrane, TGGT1_289540 appeared in strand- or filament-like structures (Fig. 1B). Western blotting confirmed the presence of a single isoform of myc-tagged protein at the predicted size of ∼100 kDa in transgenic parasites but not in a wild-type (WT) control (Fig. 1C).

To verify this unusual distribution of TGGT1_289540 in the PV, we generated rabbit polyclonal antibodies against the recombinant protein. As was the case with the myc-tagged isoform, analysis of the endogenous protein showed a filamentous distribution in the PV (Fig. 1D). Both the signal detected by immunofluorescence analysis (IFA) and a band recognized by the antibody in Western blots were absent upon the disruption of the *TGGT1_289540* locus (Fig. 1D and E).

As the *Toxoplasma* PV contains a distinct IVN of membranous tubules, we tested whether the strands were related to these structures. The formation of the IVN is dependent on GRA2, so we assessed TGGT1_289540 localization in RHΔ*gra2* parasites that lack the IVN (15). No obvious difference in filamentous TGGT1_289540 localization was observed (Fig. 1F), indicating that the filaments are distinct from, and their formation does not depend on, the IVN. We therefore named the protein strand-forming protein 1 (SFP1 here).

We hypothesized that SFP1 strands could function in transport between the parasites and the host cell. *Toxoplasma* endocytoses proteins from the host cell through an unknown mechanism (17), so we assessed whether SFP1 plays a role in the uptake of proteins from the host cell into the parasite at 3 h postinfection. As endocytosed material is rapidly digested, this can be observed only upon inhibition of cathepsin protease L function with the inhibitor morpholinurea-leucine-homophenylalanine-vinyl phenyl sulfone (LHVS) (17). Under these conditions, there was no difference in the proportions of parasites that had taken up Venus fluorescent protein from the host cell, indicating that SFP1 does not contribute to protein uptake (see Fig. S1A in the supplemental material). *Toxoplasma* upregulates host c-Myc in infected cells, a phenotype which is dependent on protein translocation from the PV into the host cell (11, 24). To test whether SFP1 plays a role in protein export into the host cell, we compared the abilities of wild-type and Δ*sfp1* parasites to upregulate host c-Myc. No differences were observed in host cell c-Myc upregulation, indicating that SFP1 does not play a role in protein export from the PV (Fig. S1B).

10.1128/mSphere.00792-20.2 FIG S1 SFP1 does not impact protein uptake or export by *Toxoplasma.* (A) Quantification of Venus in parasites liberated from host cells transfected with constructs for the expression of cytosolic Venus. Cells were infected with RH strains for 3 h in the presence of DMSO (control) or LHVS to inhibit the digestion of Venus after uptake. The proportions of parasites containing Venus are shown from duplicate experiments with >200 parasites counted per sample. (B) Upregulation of c-Myc was used as a test for protein export from the parasitophorous vacuole. HFFs were seeded under low-FBS conditions (0.5% FBS) and either stimulated for 2 h with 20% FBS or infected with RHΔ*ku80* or RHΔ*sfp1* for 18 h. DAPI and c-Myc images are shown, with examples of infected and uninfected cells indicated with white and orange arrows, respectively. Download FIG S1, PDF file, 0.1 MB. © Crown copyright 2020. 2020 Crown This content is distributed under the terms of the Creative Commons Attribution 4.0 International license.

It was recently shown that long filaments of actin extend into the PV from the parasites during *Toxoplasma* infection (25). We therefore tested whether treatment with cytochalasin D, to depolymerize actin, would disrupt SFP1 strand formation. We still saw substantial filamentation of SFP1 in the presence of cytochalasin D, indicating that actin is not important in this process (Fig. 1G).

While the presence of the IVN is not important for SFP1 filament formation, the opposite could be true, that is, that SFP1 may be important for the formation of the IVN. However, IVN formation was not affected in the absence of SFP1, as shown by transmission electron microscopy (EM) (Fig. 1H). No obvious abnormalities in the overall PV organization or PVM structure were observed in RHΔ*sfp1* parasites.

Collectively, these data indicate that SFP1, a protein that was reported to be phosphorylated after secretion, localizes to the PV and forms novel, IVN- and actin-independent filamentous structures. These structures, as suggested by our data, do not contribute to protein uptake from or protein export into the host cell.

### A second SFP1-related protein, GRA29, forms related structures.

**FIG 2:**
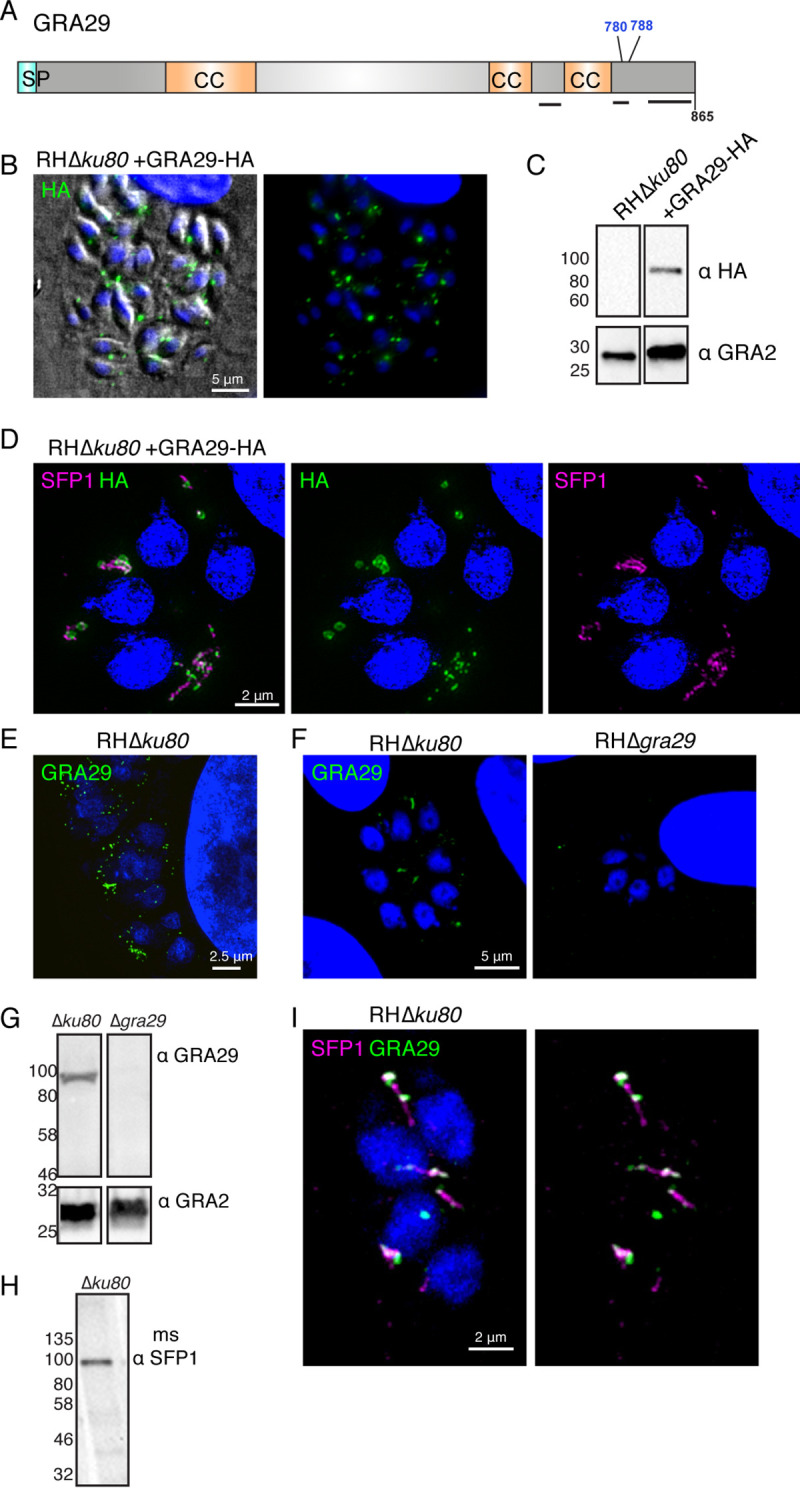
GRA29 shares homology with SFP1 and forms similar structures in the PV. (A) Schematic of GRA29 indicating predicted domains and previously identified phosphosites (shown in blue). SP, signal peptide; CC, coiled coil. Black lines show predicted unstructured regions. (B) Immunofluorescence analysis of HFFs infected with RHΔ*ku80*::GRA29-HA. Green, anti-HA antibodies; blue, DAPI. (C) Western blot analysis of RHΔ*ku80*- or RHΔ*ku80*::GRA29-HA-infected HFFs with anti-HA and anti-GRA2 antibodies to verify the expression of HA-tagged protein. (D) Structured illumination microscopy (SIM) of HFFs infected with RHΔ*ku80*::GRA29-HA. Green, anti-HA; magenta, rabbit anti-SFP1; blue, DAPI. (E) SIM analysis of RHΔ*ku80*. Green, anti-GRA29 antibodies; blue, DAPI. (F) Immunofluorescence analysis of HFFs infected with RHΔ*ku80* and RHΔ*gra29* parasites. Green, anti-GRA29; blue, DAPI. (G) Western blots of RHΔ*ku80*- and RHΔ*gra29*-infected HFFs probed with anti-GRA29 and anti-GRA2 antibodies. (H) Western blot of RHΔ*ku80*-infected HFFs probed with mouse (ms) anti-SFP1 antibodies generated against the SFP1 C terminus showing a band of the expected size. (I) SIM analysis of RHΔ*ku80*-infected HFFs. Magenta, mouse anti-SFP1; green, anti-GRA29; blue, DAPI. See also Fig. S3 in the supplemental material.

As secreted protein families can form by gene duplication and diversification, we looked for potential paralogs of SFP1 in the *Toxoplasma* genome using BLASTP. We identified a single protein (TGGT1_269690 [GRA29]) with 24% identity and a similar architecture (Fig. 2A and Fig. S2) of a signal peptide, coiled-coil domains, and, like SFP1, C-terminal phosphorylation sites that appear phosphorylated after secretion (20). GRA29 is predicted to localize to the dense granules (8) and has been shown to be secreted into the PV (26). To compare the localization of this protein with that of SFP1, we generated a hemagglutinin (HA)-tagged version, which by IFA localized to the PV, and Western blotting showed the expected size of ∼94 kDa (Fig. 2B and C). In contrast to SFP1, however, we saw smaller filamentous structures and also large puncta associated with the parasites (Fig. 2B), consistent with the localization reported by Nadipuram et al. (26). To investigate whether SFP1 and GRA29 were within the same structures, we performed superresolution microscopy using anti-SFP1 antibodies and anti-HA antibodies to localize GRA29. At this improved resolution, GRA29 puncta appeared as round spheres or doughnuts, often with SFP1 filaments associated with or branching out from them (Fig. 2D). We also observed filamentous SFP1 with smaller dots and strands of GRA29. To further investigate these unusual sphere-like structures, we performed correlative light electron microscopy (CLEM) to identify the subcellular structures. This revealed that GRA29::HA accumulates in electron-dense particles in the PV that appear to contain no membrane and resemble proteinaceous aggregates (Fig. S3).

10.1128/mSphere.00792-20.3 FIG S2 Alignment of SFP1 (TGGT1_289540) and GRA29 (TGGT1_269690) protein sequences. Predicted coiled-coil domains are shown in boldface type, and unstructured regions (of >10 residues) are underlined. Previously identified phosphosites are shown in black. Red, small, hydrophobic, aromatic, not Y; blue, acidic; magenta, basic; green, hydroxyl, amine, amide, basic; “*,” identical; “:,” conserved substitutions (same color group); “.,” semiconserved substitution (similar shapes). Download FIG S2, PDF file, 0.09 MB. © Crown copyright 2020. 2020 Crown This content is distributed under the terms of the Creative Commons Attribution 4.0 International license.

10.1128/mSphere.00792-20.4 FIG S3 GRA29-HA localizes to proteinaceous aggregates. Correlative light and electron microscopy results for HFFs infected with RHΔ*ku80* expressing GRA29-HA are shown. (A) Structured illumination microscopy highlighting the GRA29-HA structure of interest. Green, anti-HA antibody; blue, DAPI. (B) Transmission electron micrograph of the corresponding *Toxoplasma* vacuole. (C) Overlay of panels a and b after extracting high-intensity pixels from the SIM image. (D) Magnified views of panels a to c. Download FIG S3, PDF file, 0.8 MB. © Crown copyright 2020. 2020 Crown This content is distributed under the terms of the Creative Commons Attribution 4.0 International license.

To verify the GRA29::HA structures, we raised polyclonal antibodies against recombinant GRA29. With the antibodies against endogenous protein, we observed only short strands and small puncta in the PV that did not resemble the HA-tagged parasite line (Fig. 2E and F). The specificity of the antibodies was verified by Western blotting and IFA, which showed the expected molecular weight and positive IFA signal in WT but not in GRA29 knockout (KO) parasites (Fig. 2F and G). This indicates that the addition of the C-terminal HA tag disrupts GRA29 localization, inducing protein aggregates, suggesting a potentially important role of the C-terminal region. In order to colocalize endogenous nontagged SFP1 and GRA29, we generated antibodies against SFP1 peptides in mice, which showed the expected reactivity in IFAs and Western blots (Fig. 2H and I). The colocalization of SFP1 and GRA29 revealed that both proteins appear in similar structures, with GRA29 often being present at either end of SFP1 filaments (Fig. 2I).

Collectively, these data show that SFP1 and GRA29 form novel filamentous structures in the PV. C-terminal tagging of GRA29 alters its localization, either causing or stabilizing unusual structures that SFP1 can still associate with.

### GRA29 regulates SFP1 strand formation even in the absence of other *Toxoplasma* proteins.

**FIG 3:**
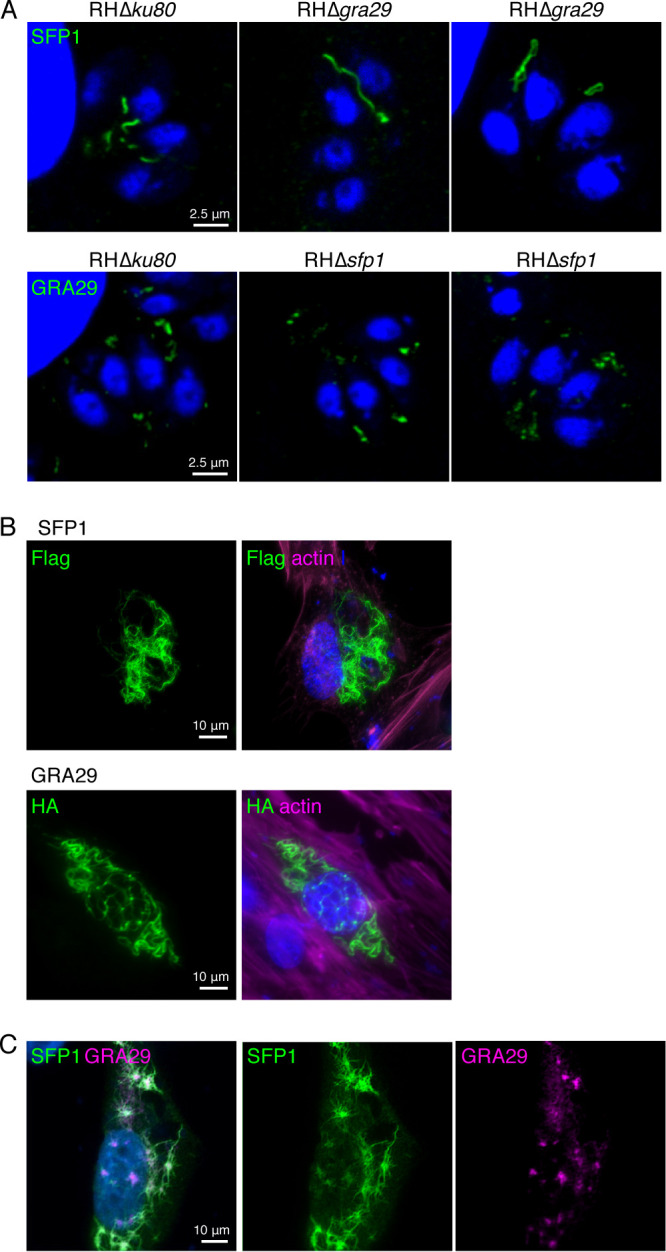
GRA29 regulates SFP1 strand formation. (A) Fewer and longer SFP1 strands form in the absence of GRA29. Immunofluorescence images of HFFs infected with RHΔ*ku80*, RHΔ*sfp1*, or RHΔ*gra29.* (Top) Rabbit anti-SFP1; (bottom) anti-GRA29. Blue, DAPI. (B) SFP1 and GRA29 form strands when ectopically expressed. Immunofluorescence images of HFFs expressing N-terminally tagged SFP1 (Flag) or GRA29 (HA). Green, anti-Flag or -HA antibodies; magenta, phalloidin to visualize F-actin; blue, DAPI. (C) HFFs coexpressing Flag-SFP1 and HA-GRA29 show SFP1 strands radiating from GRA29 foci. Immunofluorescence images of cotransfected HFFs expressing Flag-SFP1 and HA-GRA29. Green, rabbit anti-SFP1; magenta, anti-HA; blue, DAPI.

As SFP1 and GRA29 appeared to localize within the same structures, we hypothesized that they may show an interdependence. To address this, we localized SFP1 in the RHΔ*gra29* parasite line and GRA29 in the RHΔ*sfp1* parasite line using the specific antibodies raised. Whereas no alteration in GRA29 was observed in RHΔ*sfp1*, SFP1 formed fewer and longer filaments in the absence of GRA29 (Fig. 3A). To identify whether any other *Toxoplasma* proteins are required for (i) SFP1 filament formation and (ii) SFP1 filament initiation, we expressed both SFP1 and GRA29, lacking their respective signal peptides, in human foreskin fibroblasts (HFFs). Both SFP1 and GRA29 formed long filamentous structures in HFFs (Fig. 3B), often filling the complete cytoplasm and, in the case of GRA29, sometimes associated with the nucleus. When coexpressed in HFFs, GRA29 frequently formed smaller puncta and short strands, likely due to the reduced expression levels of each protein. Strikingly, SFP1 filaments radiated from GRA29 structures, suggesting that GRA29 can initiate SFP1 strand formation (Fig. 3C).

These data indicate that both SFP1 and GRA29 can each form filament-like structures independently in the absence of other *Toxoplasma* proteins. Although we were unable to confirm the interaction by coimmunoprecipitation from infected cells or with recombinant proteins (data not shown), the colocalization of SFP1 and GRA29 even in this nonphysiological context suggests that they can directly interact. Furthermore, the localization pattern observed in HFFs and the extended SFP1 filaments in parasites lacking GRA29 suggest that GRA29 regulates SFP1 strands, perhaps initiating strand formation or controlling their length.

### The SFP1/GRA29 C-terminal tail is required for strand formation.

**FIG 4:**
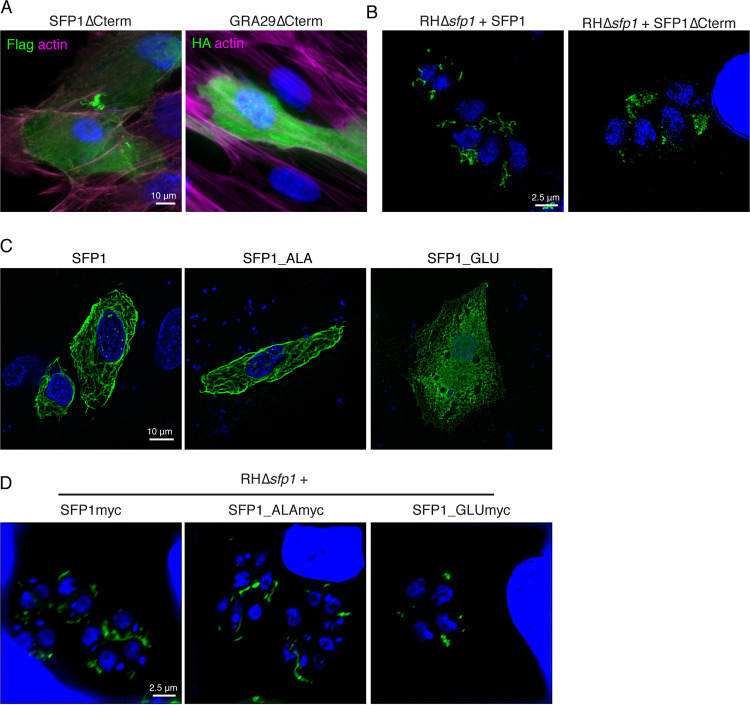
SFP1 and GRA29 C termini regulate strand formation. (A) Immunofluorescence analysis of HFFs expressing truncated Flag-SFP1 or HA-GRA29 lacking its disordered C terminus. Green, anti-Flag or -HA antibodies; magenta, phalloidin to visualize F-actin; blue, DAPI. (B) SIM analysis of HFFs infected with RHΔ*sfp1* expressing SFP1-myc or SFP1ΔCterm-myc. Green, rabbit anti-SFP1 antibodies; blue, DAPI. (C) SFP1 phosphomutants (preventing phosphorylation) do not disrupt strand formation. Immunofluorescence analysis of HFFs expressing Flag-SFP1 or Flag-SFP1 with the C-terminal phosphosites mutated to Ala or Glu. Green, anti-Flag antibodies; blue, DAPI. (D) Immunofluorescence analysis of HFFs infected with RHΔ*sfp1* expressing myc-tagged SFP1, SFP1_ALA, or SFP1_GLU. Green, anti-myc antibodies; blue, DAPI.

The change of the localization of GRA29 upon C-terminal HA tagging and the cluster of phosphorylation sites in the C termini of SFP1 and GRA29 suggested that the C-terminal tail may play an important role in the regulation of strand formation. To address this, we expressed truncated SFP1 and GRA29 lacking the unstructured C-terminal region in HFFs. In contrast to forming filaments under these conditions, both proteins appeared distributed throughout the HFF cytoplasm, with sparse foci of aggregation observed (Fig. 4A). This strongly suggested that the C terminus of the protein plays an important role in filament formation. Consistently, during *Toxoplasma* infection with RHΔ*sfp1*+SFP1Δ*Ct*, there was a lack of typical SFP1 strand formation in the PV (Fig. 4B). However, SFP1Δ*Ct* appeared aggregated within the PV rather than dispersed, suggesting that other PV-resident proteins impact SFP1 distribution.

Having established that the C terminus of SFP1 is important for its subcellular organization, we asked whether the phosphorylation of SFP1 is important for filament formation. To investigate this, we first turned to the expression of phosphomutants in HFFs, in which all phosphorylation sites in the SFP1 C terminus were mutated to alanine (SFP1_ALA), and phosphomimetics, in which all phosphorylation sites were mutated to glutamic acid (SFP1_GLU). While SFP_ALA mutants displayed normal filaments in HFFs, mimicking phosphorylation led to an inability of SFP1 to form filaments. This indicates that phosphorylation may be a negative regulator of filament formation (Fig. 4C). This was replicated when SFP1_ALA and SFP1_GLU were expressed in RHΔ*sfp1* parasites (Fig. 4D), where extended strands were observed with phosphomutant but not phosphomimetic mutations.

If phosphorylation was preventing filament formation, we hypothesized that SFP1 expressed in HFFs should not be phosphorylated. To verify that assumption, we immunoprecipitated SFP1 from HFFs and analyzed the proteins by mass spectrometry (MS). Despite the presence of many nonmodified peptides from SFP1, we observed no phosphopeptides (data not shown), indicating that, indeed, SFP1 is not phosphorylated by human kinases when expressed in HFFs.

Collectively, these data show that the C termini of SFP1 and GRA29 are important for their regulation and that the phosphorylation of SFP1 appears to be a negative regulator, with the nonphosphorylated protein forming strands.

### SFP1 and GRA29 are dispersed and do not form strands in chronic-stage cysts.

**FIG 5:**
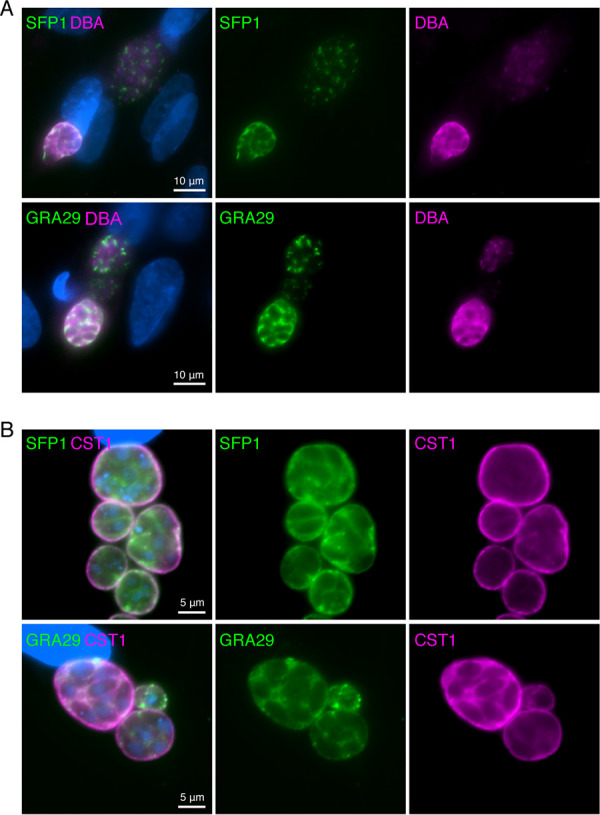
SFP1 and GRA29 disperse in chronic-stage cysts. (A) Immunofluorescence analysis of HFFs infected with PruΔ*hxgprt* for 40 h. Green, rabbit anti-SFP1 or anti-GRA29; red, DBA to stain the cyst wall; blue, DAPI. (B) Immunofluorescence analysis of PruΔ*hxgprt*-infected HFFs grown under bradyzoite-inducing conditions for 3 days. Green, rabbit anti-SFP1 or anti-GRA29; magenta, anti-CST1 to visualize the cyst wall; blue, DAPI.

To determine whether SFP1 and GRA29 are required *in vivo*, we generated gene KOs in the type II strain Pru and verified gene disruption by Western blotting (Fig. S4A). Mice infected with PruΔ*sfp1* and PruΔ*gra29* parasites succumbed to infection similarly to WT PruΔ*ku80* parasites, indicating that neither protein is essential for parasite survival *in vivo* (Fig. S4B and C). During our experiments with the Pru strains, we observed that in some vacuoles, SFP1 and GRA29 appeared distributed throughout the PV space instead of forming characteristic strands (Fig. 5A). Type II strains differ from the type I RH strain in that they more frequently convert to slow-growing bradyzoites in tissue culture. Bradyzoite cysts can be distinguished from tachyzoite vacuoles using fluorescently labeled lectin (Dolichos biflorus agglutinin [DBA]) that binds to the cyst wall (27). Parasites with a disperse SFP1/GRA29 pattern also stained positive for the cyst wall marker (Fig. 5A). Spontaneous conversion to bradyzoites occurs at low levels in standard tissue culture, but high levels of conversion can be induced by pH stress (28). Under bradyzoite-inducing conditions, parasite vacuoles were positive for the cyst wall marker CST1 and showed disperse SFP1 and GRA29, indicating that both proteins undergo a major change in localization between the tachyzoite and bradyzoite stages (Fig. 5B).

10.1128/mSphere.00792-20.5 FIG S4 SFP1 and GRA29 are not essential for parasite virulence *in vivo*. (A) Western blots verifying the loss of SFP1 and GRA29 in PruΔ*ku80* gene knockouts. Lysates of HFFs infected with PruΔ*ku80*, PruΔ*sfp1*, or PruΔ*gra29* were probed with anti-SFP1, anti-GRA29, anti-GRA1, and anti-GRA2 antibodies. (B and C) C57BL/6 mice were injected with 300,000 (B) or 50,000 (C) tachyzoites of PruΔ*ku80*, PruΔ*sfp1*, or PruΔ*gra29*, and their survival was monitored. Only mice shown to be infected by BLI with a signal above background levels were included in the survival analysis. No significant difference was observed with a total of >6 mice per strain (for panel b, *n* = 4 mice for PruΔ*ku80*, *n* = 3 mice for PruΔ*sfp1*, and *n* = 3 mice for PruΔ*gra29*; for panel c, *n* = 2 mice for PruΔ*ku80*, *n* = 4 mice for PruΔ*sfp1*, and *n* = 4 mice for PruΔ*gra29*). Download FIG S4, PDF file, 0.2 MB. © Crown copyright 2020. 2020 Crown This content is distributed under the terms of the Creative Commons Attribution 4.0 International license.

### Secreted proteins are differentially phosphorylated in the chronic stage.

**FIG 6:**
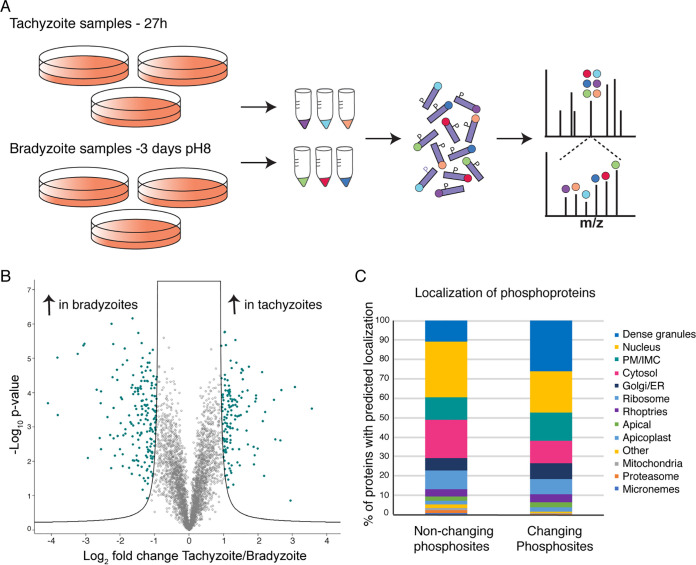
Secreted proteins are differentially phosphorylated in acute and chronic stages. (A) Schematic of the quantitative phosphoproteomics experiment. Peptides from triplicate tachyzoite and bradyzoite samples were labeled with tandem mass tags (TMTs), enriched for phosphopeptides, and analyzed by mass spectrometry. (B) Volcano plot showing differential phosphorylation between tachyzoite and bradyzoite conditions after normalization to protein abundance. Each circle represents a phosphosite, and those that change significantly (FDR, 1%; −0.9 > log_2_-fold change > 0.9) are shown in green. (C) Dense granule proteins are enriched in the subset of proteins that show significantly changing phosphorylation between tachyzoites and bradyzoites. The chart shows the predicted localization of phosphoproteins using LOPIT (8). PM, plasma membrane; IMC, inner membrane complex; ER, endoplasmic reticulum.

We hypothesized that increased phosphorylation of SFP1 and GRA29 under bradyzoite conditions could lead to their dispersion. This phenomenon of redistribution in the bradyzoite cyst has been described previously for other GRAs (22, 29), and so we wanted to determine if these were also differentially phosphorylated. We therefore used comparative phosphoproteomics to determine differences in the phosphorylation of SFP1 and GRA29 and, more broadly, between acute (tachyzoite) and chronic (bradyzoite) conditions. Triplicate samples of *Toxoplasma* parasites were grown under tachyzoite conditions for 27 h or bradyzoite conditions for 3 days (to give comparable numbers of parasites per vacuole) (Fig. 6A). We performed quantitative mass spectrometry to compare protein and phosphosite abundances under the two conditions using tandem mass tags (TMTs) as described previously (30, 31). First, we analyzed differential protein abundances and identified 171 differentially regulated proteins, with 103 proteins with higher abundances in bradyzoites and 68 with higher abundances in tachyzoites. As expected, known bradyzoite markers such as BAG1, ENO1, LDH2, and MAG1 were more abundant in the bradyzoite samples than in the tachyzoite samples (Data Set S1 and Fig. S5).

10.1128/mSphere.00792-20.6 FIG S5 Differential protein expression under tachyzoite and bradyzoite conditions. Volcano plots show differential protein abundances under tachyzoite and bradyzoite conditions. Each circle represents a protein, and those that change significantly between conditions (FDR, 1%; 0.6 > log_2_-fold change > 0.6) are shown in green. The known bradyzoite markers BAG1, MAG, ENO1, and LDH2 are labeled and are enriched in the bradyzoite samples. Download FIG S5, PDF file, 0.05 MB. © Crown copyright 2020. 2020 Crown This content is distributed under the terms of the Creative Commons Attribution 4.0 International license.

10.1128/mSphere.00792-20.8 DATA SET S1 TMT-based quantification of differential phosphorylation and protein abundances in tachyzoites and bradyzoites. (Sheet 1) Quantified phosphosites with proteome data. TMT reporter intensities obtained via MS2- and MS3-based acquisition methods were averaged. The data were filtered to remove common contaminants and IDs originating from reverse decoy sequences. The log_2_ values of reporter intensities for tachyzoites and bradyzoites were then determined and normalized to the available proteome data to reflect changes in phosphorylation and not in protein abundance. To identify significantly changing phosphosites, a modified *t* test with permutation-based FDR statistics was performed (250 permutations; FDR of 0.01; log_2_-fold change threshold of ±0.9 [3 times the median absolute deviation]) on normalized tachyzoite (*n* = 3) and bradyzoite (*n* = 3) samples. The raw data were searched against a T. gondii fasta file consisting of TGGT1, TGME49, and TGVEG protein IDs. TGME49 IDs and the corresponding site information are given when an exact sequence match was identified. TGGT1/TGVEG IDs are included where no match to the TGME49 sequence was identified. (Sheet 2) Differentially regulated phosphosites. (Sheet 3) Quantified proteome. The data were filtered to remove common contaminants and protein IDs originating from reverse decoy sequences and identified only by site. The log_2_ values of reporter intensities were then determined and normalized by median subtraction. To identify proteins with differential abundances, a modified *t* test with permutation-based FDR statistics was performed (250 permutations; FDR of 0.01; log_2_-fold change threshold of ±0.6 [3 times the median absolute deviation]) on tachyzoite (*n* = 3) and bradyzoite (*n* = 3) samples. Protein IDs are given as TGME49. TGGT1/TGVEG IDs are included where no match to the TGME49 sequence was identified. (Sheet 4) Differentially regulated proteome. Download Data Set S1, XLSX file, 2.4 MB. © Crown copyright 2020. 2020 Crown This content is distributed under the terms of the Creative Commons Attribution 4.0 International license.

In the phosphopeptide-enriched samples, we quantified 7,650 phosphorylation sites on 2,235 *Toxoplasma* proteins. For 3,730 of the phosphorylation sites, we also obtained quantitative proteome data to which phosphorylation site changes were normalized. This allowed us to assess true differential phosphorylation rather than changes originating from differential protein abundances between conditions. After this normalization step, we identified 337 phosphorylation sites that were significantly different between tachyzoite (144 sites more phosphorylated) and bradyzoite (193 sites more phosphorylated) stages (Fig. 6B and Data Set S1). The 337 phosphorylation sites are found on 170 proteins, 51 of which are predicted to be secreted (ROPs or GRAs in the LOPIT data set [8]). While GRAs made up 11% of proteins with nonchanging phosphosites, they represented 26% of differentially phosphorylated proteins (Fig. 6C). This enrichment of GRAs in the subset of proteins that are differentially phosphorylated between tachyzoite and bradyzoite conditions indicates that these are indeed subject to extensive differential phosphorylation during stage conversion.

In this experiment, we also identified phosphosites corresponding to the C termini of SFP1 and GRA29, but they did not significantly change between conditions (although a previously undetected N-terminal site on GRA29 was detected as more phosphorylated in bradyzoites). This indicates that while mimicking phosphorylation disrupts SFP1 strands in the PV in tachyzoites, this modification is not sufficient for the dispersion observed in the forming cyst. However, the GRAs that were detected as differentially phosphorylated included IVN-localized GRA2, GRA4, GRA6, and GRA12 and PV membrane GRA1 and GRA5 (Table 1). We also detected differential phosphorylation of the cyst wall proteins CST1, CST3, CST4, and CST6. Furthermore, of the 38 recently identified putative cyst wall components (32), 24 were differentially phosphorylated, suggesting that phosphorylation could play an important role in the formation of this structure.

**TABLE 1:** Differential phosphorylation of predicted dense granule proteins between tachyzoites and bradyzoites^*a*^

Protein ID	Protein annotation/name from ToxoDB comments		Proteome T vs B LFC
	Phosphosite position	T vs B LFC	
TGME49_275440	Dense granule protein GRA6		−0.94
	112	1.45	
	118	0.98	
	128	2.12	
	132	1.94	
	133	1.23	
	134	1.38	
	218	1.74	

TGME49_213067	Hypothetical protein/GRA36		−0.74
	67	−1.80	
	76	−1.01	
	132	−1.00	
	133	−1.03	
	171	−1.20	

TGME49_290700	Hypothetical protein/GRA25		−0.68
	66	−1.22	
	248	1.33	
	275	1.18	
	278	1.28	
	280	1.11	

TGME49_260520	Hypothetical protein/GRA53/CST6		−0.56
	187	−1.47	
	511	−1.91	
	513	−1.49	

TGME49_267740	Hypothetical protein		−0.51
	195	1.67	
	196	1.08	

TGME49_312330	Hypothetical protein		−0.44
	1790	−2.19	
	1799	−1.98	
	1801	−2.56	
	1802	−1.70	

TGME49_263220	Rhoptry kinase family protein ROP21		−0.43
	246	−1.07	

TGME49_220240	Hypothetical protein/GRA31		−0.42
	176	1.06	
	177	1.18	
	424	1.15	

TGME49_240060	Hypothetical protein/TgIST		−0.41
	486	1.03	
	550	1.56	

TGME49_247440	Hypothetical protein/GRA33		−0.37
	68	−0.98	

TGME49_236890	Hypothetical protein/GRA37		−0.33
	83	−0.99	
	90	1.70	
	91	1.12	
	94	1.45	
	95	1.05	
TGME49_217680	Hypothetical protein/GRA57		−0.30
	150	−2.25	
	613	0.98	
	630	−1.33	
	632	−1.10	
	2034	−1.42	
	2039	−1.22	

TGME49_288650	Dense granule protein GRA12		−0.24
	415	−1.94	

TGME49_269690	Hypothetical protein/GRA29		−0.24
	46	−1.49	

TGME49_203310	Dense granule protein GRA7		−0.22
	41	1.23	
	90	1.17	
	130	−1.64	
	227	1.20	

TGME49_227620	Dense granule protein GRA2		−0.22
	56	−2.08	
	72	1.41	
	76	2.14	

TGME49_230705	Hypothetical protein/GRA51/CST3		−0.20
	72	−0.97	

TGME49_289380	Hypothetical protein/GRA39		−0.16
	326	−1.55	

TGME49_270250	Dense granule protein GRA1		−0.09
	186	−0.96	

TGME49_254720	Dense granule protein GRA8		−0.01
	198	1.10	

TGME49_200360	Hypothetical protein		0.01
	152	1.23	

TGME49_270320	Protein phosphatase 2C domain-containing protein		0.02
	149	−1.21	
	150	−1.12	
	529	−1.38	

TGME49_311230	Hypothetical protein		0.09
	3393	1.54	
	3456	1.27	
	3457	1.12	
	4646	1.16	
	4597	1.04	

TGME49_233010	Cell cycle-associated protein kinase ERK7, putative		0.12
	631	1.14	

TGME49_201130	Rhoptry kinase family protein ROP33		0.21
	394	1.05	

TGME49_225160	Hypothetical protein		0.22
	391	−1.12	
TGME49_244530	Hypothetical protein		0.24
	219	−1.37	
	384	−1.37	
	548	−1.21	
	552	−1.64	

TGME49_247530	Hypothetical protein		0.40
	53	1.12	

TGME49_286450	Dense granule protein GRA5		0.43
	31	−1.63	
	33	−1.29	
	105	−1.07	

TGME49_278770	Hypothetical protein		0.50
	220	−1.11	

TGME49_275470	Dense granule protein GRA15		0.58
	521	1.10	

TGME49_231960	Omega secalin, putative/GRA28		0.65
	1208	1.89	
	1789	1.19	

TGME49_310780	Dense granule protein GRA4		0.65
	251	−1.63	
	260	−1.62	
	278	−1.19	
	285	−1.19	

TGME49_279100	Hypothetical protein/MAF1a		0.71
	48	−1.39	
	62	−1.20	

a Shown is a subset of the data in Data Set S1 in the supplemental material showing proteins predicted to localize to the dense granules with a log_2_-fold change (LFC) in protein abundance of <1. Log_2_-fold changes for the proteome and the normalized LFCs for each phosphosite are shown. Negative scores indicate enrichment in bradyzoites. For full details, see Data Set S1. T, tachyzoites; B, bradyzoites.

It is worth noting that we also observed differential phosphorylation on proteins important for DNA regulation. This includes the AP2 domain transcription factors AP2VIII-2 and AP2XII-4 and 32 predicted nuclear proteins. These may be important for transcriptional regulation during stage conversion, but their role is not further discussed here.

Collectively, these data show that SFP1 and GRA29 undergo a dramatic change in localization between tachyzoite and bradyzoite conversion but that their phosphorylation is transient or not required for this transition, at least at the experimental time point selected here. Furthermore, we show widespread changes in phosphorylation states in a substantial number of secreted proteins, indicating the activity of stage-specific kinases and phosphatases in regulating PV-resident proteins.

## DISCUSSION

Here, we have analyzed the localization of two secreted phosphoproteins, SFP1 and GRA29, that relocalize during stage conversion to bradyzoites. Both proteins form stranded structures in the tachyzoite PV that are distinct from the IVN and actin filaments previously observed. We did not identify a function of SFP1 and GRA29 during the lytic cycle or *in vivo* infections, preventing us from assessing the impact of their phosphorylation on *Toxoplasma* biology. It could be that the proteins have a subtle effect on growth that we have not been able to measure or that they are important in another host species, parasite stage, or genetic background that we have not tested here. However, neither SFP1 nor GRA29 showed a reduction in fitness *in vivo* (33) in our recent more sensitive *in vivo* CRISPR screen, supporting a nonessential role under these conditions.

We show the importance of the C termini of both proteins for strand formation. Using mutational analysis, we show that SFP1 phosphorylation is not required for filament formation and that mimicking phosphorylation disrupts filament formation. As such, mimicking phosphorylation mirrored the phenotype observed with the removal of the C-terminal tail. Phosphorylation negatively regulates the intrinsic behavior of SFP1 and abrogates its ability to form strands. However, as both SFP1 and GRA29 were identified to be phosphorylated after secretion into the PV (20), where, as we show here, they form fibril-like structures, we assume that their phosphorylation is a dynamic process, potentially leading to the regulation of their length and location. It could also be that phosphorylated and nonphosphorylated SFP1 and GRA29 localize to different structures in the PV. How both proteins relocalize during stage conversion is unclear. While mimicking phosphorylation appears sufficient to promote the dispersal of SFP1 HFFs, this is not observed when expressed in tachyzoites, where aggregation is observed. This indicates that other proteins or posttranslational modifications are involved in SFP1 regulation in parasites upon stage conversion.

Recently, the PV-localized kinase WNG1/ROP35 was shown to phosphorylate several GRAs, including GRA6, and to regulate their membrane association (21). As many GRAs form complexes during trafficking (34), WNG1 phosphorylation is hypothesized to release them from association with a chaperone, freeing them to associate with membranes. As such, WNG1-dependent phosphorylation of GRAs was shown to promote their normal behavior and localization. We now propose that in addition to WNG1 phosphorylation promoting the release and membrane association of GRAs, the phosphorylation of secreted proteins can negatively regulate their behavior. In this way, the parasite could use phosphorylation by kinases within the PV lumen to regulate PV-resident proteins. This would allow the parasite to tailor its niche and respond to differing conditions or requirements.

Several secreted kinases localize within the PV lumen and are upregulated during the chronic stages of infection (for example, ROP21, ROP27, ROP28, and, indeed, WNG1/ROP35). Importantly, secreted kinases have been shown to contribute to cyst formation in mice, indicating that phosphorylation within the PV/forming cyst is required for cyst development. ROP21/27/28 contribute to the parasite establishing chronic infection, but their function and targets remain unknown (35). Additionally, a triple knockout of ROP38/29/19 showed a severe defect in cyst formation (36). Here, we expand our knowledge of cyst formation by reporting the differential phosphorylation of proteins within the forming cyst. Our data also identify phosphorylation that is reduced upon stage conversion. The expression of many ROP family kinases is reduced in the chronic stage of infection, which could account for some changes, but it is likely that secreted phosphatases are contributing to the differential phosphorylation. The necessity of deleting several ROPs to observe strong phenotypic effects on bradyzoites may reflect that the system is flexible with a high degree of redundancy. Alternatively, the combined effect of multiple kinases could allow the parasite to integrate several signals leading to stage differentiation.

PV-resident GRAs relocalize as the parasite converts to the chronic stage and remodels the PV to form a cyst. Guevara et al. recently described the dynamic nature of this relocalization over the course of cyst formation (for GRA1, -2, -4, -6, -9, and -12) (22). Furthermore, they showed that IVN-localized GRAs were required for normal formation of the cyst wall. Here, we show that these GRAs, with the exception of GRA9 (which is upregulated in bradyzoites), are differentially phosphorylated in the acute and chronic stages, suggesting that phosphorylation could play a role in their regulation. Interestingly, GRA2 showed phosphorylation sites that were enriched under each condition; two sites within a predicted amphipathic helix were more phosphorylated under tachyzoite conditions, while a third site was more phosphorylated in the bradyzoite samples. It is tempting to speculate that dual phosphorylation within the polar side of the amphipathic helix (a1) of GRA2 could alter its membrane binding. Further work assessing the phosphorylation of secreted proteins over time is required to decipher the impact of this modification and the role of both kinases and phosphatases in remodeling the PV. However, the regulation of proteins after parasite secretion adds an intriguing extra layer of complexity to pathogen effector biology and the establishment of *Toxoplasma* chronic infection.

## MATERIALS AND METHODS

### Cell culture and parasite strains.

Primary human foreskin fibroblasts (HFFs) (ATCC) were maintained in Dulbecco’s modified Eagle’s medium (DMEM) with 4.5 g/liter glucose and GlutaMAX-1 (Gibco) supplemented with 10% fetal bovine serum (FBS) at 37°C with 5% CO_2_. T. gondii RHΔ*ku80*::diCre (37), RHΔ*gra2* (38), PruΔ*ku80* (39), and PruΔ*hxgprt* (gift from Dominique Soldati) (40) parasites were maintained by growth in confluent HFFs and passaged every 2 to 3 days. RH strains were seeded 24 h, and Pru strains were seeded 48 h, before transfections or infection experiments.

### Sequence analysis.

Gene sequences and predicted protein localizations (LOPIT data set) were obtained from ToxoDB. Alignments were generated using ClustalW, and schematics were generated using DOG2.0 (41). Protein sequences were analyzed by Multicoil2 (42) and Globplot (43) to identify coiled-coil and unstructured regions, respectively.

### Plasmid construction.

All primers are shown in Table S1 in the supplemental material. Vectors were generated by inverse PCR using KOD hot-start polymerase (Novagen) or by Gibson assembly in a homemade reaction master mix (100 mM Tris-Cl [pH 7.5], 50 mg/ml polyethylene glycol 8000 [PEG 8000], 10 mM MgCl_2_, 10 mM dithiothreitol [DTT], 0.2 mM [each] four deoxynucleoside triphosphates [dNTPs], 1 mM NAD with 0.8 U T5 exonuclease [NEB], 0.5 U Phusion DNA polymerase [NEB], and 80 U *Taq* DNA ligase [NEB]). Gibson reaction mixtures were incubated for 60 min at 50°C. All vectors were verified by sequencing. *sfp1* and *gra29* sequences were amplified from *Toxoplasma* genomic DNA (gDNA) prepared from 1E7 RHΔ*ku80*::diCre parasites using a Qiagen blood and tissue DNA extraction kit, unless otherwise stated.

10.1128/mSphere.00792-20.7 TABLE S1 Oligonucleotides. Download Table S1, DOCX file, 0.01 MB. © Crown copyright 2020. 2020 Crown This content is distributed under the terms of the Creative Commons Attribution 4.0 International license.

To generate pSFP1, the TGGT1_289540 promoter and coding sequence were amplified using primers 1 and 2 and inserted into HindIII/PacI-digested pGRA (13). A C-terminal myc tag was inserted by inverse PCR using primers 3 and 4. pSFP1ΔCterm, which lacks residues 863 to 937, was generated by inverse PCR on pSFP1 with primers 4 and 5. To generate phosphomutant versions of pSFP1, initially, a silent ApaI site was inserted into pSFP1 (at nucleotide [nt] 2529) by inverse PCR with primers 6 and 7. Four-hundred-base-pair synthetic DNA fragments (IDT) (Text S1) encoding the SFP1-myc C terminus or versions with Ser/Thr-to-Ala or Ser/Thr-to-Glu mutations were mixed with ApaI/PacI-digested pSFP1 ApaI in Gibson reaction mix. The sites mutated to Ala or Glu were S862, S868, S871, S887, S891, S897, S906, S913, S924, and T933. pGRA29-HA was generated by amplifying the GRA29 promoter and coding sequence using primers 8 and 9 and inserting the amplicon into HindIII/NcoI-digested pGRA.

10.1128/mSphere.00792-20.1 TEXT S1 Synthetic DNA sequences used in this study. Download Text S1, DOCX file, 0.02 MB. © Crown copyright 2020. 2020 Crown This content is distributed under the terms of the Creative Commons Attribution 4.0 International license.

For pcDNA-NTAP-Flag SFP1 constructs, the SFP1 coding sequence excluding the signal peptide (amino acids 1 to 53) was amplified with primers 10 and 11 for full-length or primers 10 and 12 for C-terminally truncated (lacking amino acids 863 to 937) versions. For phosphosite mutant versions, primers 10 and 11 were used with pSFP1_ALA or pSFP1_GLU as the template. All amplicons were inserted into NotI/EcoRI-digested pcDNA-NTAP, a pcDNA3.1 derivative for expression with a 3× Flag tag, a tobacco etch virus (TEV) cleavage site, and a calmodulin binding peptide. For the pcDNA-HA-GRA29 construct, GRA29 lacking its predicted signal peptide (residues 1 to 25) was initially amplified with primers 13 and 14 and inserted into NotI/EcoRI-digested pcDNA-NTAP. The Flag tag was removed, and an N-terminal HA tag was inserted by inverse PCR with primers 15 and 16. pcDNA-GRA29ΔCterm, lacking residues 771 to 865, was generated by inverse PCR with primers 17 and 18.

Guide RNA (gRNA) sequences targeting the 5′ and 3′ ends of *sfp1*/*gra29* were inserted into pSAG1::CAS9-U6::gUPRT (44) by inverse PCR with primers 19 and 20 to 23. Dual gRNA pSAG1::CAS9 vectors were generated by amplifying the 3′ gRNA expression locus with primers 24 and 25 and inserting the region into XhoI/PacI-digested pSAG1::CAS9 carrying the 5′ gRNA.

To generate the SFP1 bacterial expression plasmid pET-28a-SFP1, SFP1 lacking its amino-terminal signal peptide was amplified using primers 26 and 27. The amplicon was cloned into the BamHI- and NdeI-digested pET-28a(+) plasmid (Merck).

### Parasite transfection.

Parasites were released from HFFs by syringe lysis, pelleted at 650 × *g*, and resuspended in cytomix (10 mM KPO_4_, 120 mM KCl, 0.15 mM CaCl_2_, 5 mM MgCl_2_, 25 mM HEPES, 2 mM EDTA). Approximately 5E6 parasites were combined with 40 μg DNA in 400 μl cytomix in a 2-mm cuvette and electroporated using a Bio-Rad Gene Pulser Xcell instrument at 1,500 V and 25 μF. Repair templates were linearized by overnight digestion, purified by phenol-chloroform precipitation, and resuspended in H_2_O. At 24 h posttransfection, 50 μg/ml mycophenolic acid (Merck) and xanthine (Sigma) (M/X) was added to select for transfectants.

### Generation of parasite lines.

RHΔ*ku80* lacking SFP1 was generated by transfecting parasites with pSAG1-CAS9gSFP1 targeting the 5′ end of the gene (starting 84 nt after ATG) with a 183-nt repair template to remove the protospacer-adjacent motif (PAM) site and introduce stop codons (Text S1). Parasites expressing the transfected Cas9-green fluorescent protein (GFP) were sorted by flow cytometry into 96-well plates containing HFF monolayers using a BD Influx cell sorter (BD Biosciences). This line was used for complementing pSFP1myc constructs. Alternatively, *gra29* and *sfp1* were disrupted in the RHΔ*ku80* and PruΔ*ku80* backgrounds by cotransfecting a double gRNA pCAS9 vector targeting the 5′ and 3′ ends of the gene and a synthetic repair construct with 500-nt homology arms and a hypoxanthine-xanthine-guanine phosphoribosyl transferase (HXGPRT)-thosea asigna virus 2A (T2A)-mCherry cassette (GeneArt; Life Technologies). Transfectants were selected by the addition of M/X, and clones were screened by immunofluorescence and verified by Western blotting.

### Antibody generation.

N-terminally 6×His-tagged SFP1 recombinant protein was expressed from pET-28a-SFP1 in Escherichia coli BL21 cells. His-SFP1 was purified using Ni-nitrilotriacetic acid (NTA) affinity purification under native conditions according to the standard manufacturer’s protocol (Qiagen). Recombinant His-SFP1 was used to immunize female New Zealand White rabbits (Covalab) for the generation of polyclonal antibodies. To generate the mouse anti-SFP1 antibody, phosphopeptides corresponding to the C terminus of SFP1 were conjugated to keyhole limpet hemocyanin and used to immunize 4 mice (Covalab). Day 53 sera showed reactivity against SFP1. While mice were immunized with phosphorylated isoforms, the antibodies recognize nonphosphorylated peptides.

The following SFP1 peptides were used for immunization (residues in boldface type are phosphorylated): NH_2_-C-GSVA**S**GFRG-CONH_2_, NH_2_-C-GFRG**S**MA**S**GLFP-CONH_2_, NH_2_-C-LRGA**S**VAG**S**LGG-CONH_2_, NH_2_-C-GGVG**S**RLGG-CONH_2_, and NH_2_-C-FAGA**S**MGRG-CONH_2_.

### Immunoprecipitation.

HFFs were infected with RHΔ*ku80* or RHΔ*ku80*+SFP1::myc in a T25 flask for 24 h before lysis in 1 ml radioimmunoprecipitation assay (RIPA) buffer (Thermo Scientific) supplemented with protease (complete mini; Roche) inhibitors. Samples were clarified by centrifugation and incubated with anti-myc tag antibody-conjugated agarose (catalog no. 16-219; Millipore) for 3 h. The nonbound fraction was removed, and the proteins were precipitated with acetone at −20°C. Agarose bead complexes were washed in RIPA buffer followed by Tris-buffered saline, and proteins were eluted by boiling in Laemmli buffer (60 mM Tris-HCl [pH 6.8], 1% sodium dodecyl sulfate [SDS], 5% glycerol, 5% β-mercaptoethanol, 0.01% bromophenol blue).

### Western blotting.

*Toxoplasma* lysates or immunoprecipitation (IP) samples were separated by SDS-polyacrylamide gel electrophoresis (PAGE), transferred onto nitrocellulose (Bio-Rad), and probed with appropriate antibodies in phosphate-buffered saline (PBS) with 3% milk and 0.1% Tween 20 (Sigma-Aldrich). Rabbit and mouse anti-SFP1 sera were used at a dilution of 1:1,000, rabbit anti-GRA29 serum was used at 1:1,000 (33), mouse anti-GRA1 was used at 1:1,000 (gift from Jean-Francois Dubremetz), mouse anti-GRA2 was used at 1:1,000 (catalog no. BIO.018.5; Biotem), mouse anti-Myc was used at 1:1,000 (catalog no. 05-724; Millipore), and rat anti-HA was used at 1:1,000 (catalog no. 11867423001; Roche). After washing, the blots were incubated with horseradish peroxidase (HRP)-conjugated secondary antibodies, anti-rabbit at 1:20,000 (catalog no. 474-1506; Insight Biotechnology), anti-mouse at 1:3,000 (catalog no. 474-1506; Insight Biotechnology), or anti-rat at 1:4,000 (catalog no. 629520; Life Technologies). The blots were visualized using ECL Western blotting detection reagent (GE Healthcare) for chemiluminescence imaging on an Amersham Imager 600 instrument (GE Healthcare).

### Parasite infections.

For wide-field, confocal, or EM imaging, HFFs were seeded on 13-mm no. 1.5 coverslips in 24-well plates. Confluent monolayers were infected at a multiplicity of infection (MOI) of 1 for 16 to 24 h. For cytochalasin D treatment, infected cells were incubated with 1 μM cytochalasin D or dimethyl sulfoxide (DMSO) for 1 h before fixation. Samples were washed with PBS before fixation in 3% formaldehyde (FA) for 15 min. For EM analysis, infected cells were fixed in 4% FA for 15 min followed by 2.5% glutaraldehyde–4% FA for 30 min and storage in 1% FA.

### Conversion to bradyzoites.

To induce parasite switching to bradyzoites, HFF monolayers were infected at an MOI of 0.5 for 3.5 h and then washed four times with PBS before the addition of switch medium (RPMI 1640 [catalog no. R4130; Sigma] with 1% FBS [pH 8.1]) and incubation at 37°C with ambient CO_2_ for 3 days. Cells were fixed in 3% FA, quenched in 50 mM ammonium chloride, and permeabilized in a solution containing 0.2% Triton X-100, 0.1% glycine, and 0.2% bovine serum albumin (BSA) in PBS for 20 min on ice.

### Intracellular fluorescent-protein ingestion assay.

Intracellular fluorescent-protein ingestion was assessed as described previously by McGovern et al. (45). Briefly, 70 to 80% confluent CHO‐K1 cells were transfected in 35-mm dishes using X-TremeGENE 9 transfection reagent (catalog no. 6365787001; Roche). Each dish was transfected with 2 μg of the pcDNA 3.1 Venus plasmid using a 3:1 ratio of plasmid to transfection reagent in Opti‐MEM (catalog no. 31985062; Gibco) and a total final volume of 200 μl. The CHO K1 cells were then incubated overnight at 37°C and synchronously infected with T. gondii parasites using the Endo buffer method at 18 to 24 h posttransfection (46). T. gondii parasites were treated with 1 μM LHVS or an equal volume of DMSO for 48 h prior to synchronous invasion, replacing the LHVS- and DMSO-treated media every 6 to 18 h, and also during the 3-h incubation period after synchronous invasion. T. gondii parasites were purified at 3 h postinvasion as previously described (17), pelleted by centrifugation at 1,000 × *g* for 10 min at 4°C, and subjected to a protease protection assay by resuspension in 250 μl of freshly prepared 1 mg/ml pronase (catalog no. 10165921001; Roche)–0.01% saponin–PBS at 12°C for 1 h. The parasites were then pelleted by centrifugation at 1,000 × *g* for 10 min and washed 3 times in ice-cold PBS before depositing them on Cell‐Tak (catalog no. 354240; Corning)-coated chamber slides. Parasites were fixed with 4% paraformaldehyde and imaged with an AxioCam MRm camera‐equipped Zeiss Axiovert Observer Z1 inverted fluorescence microscope. Ingestion of host cytosolic Venus was scored manually as Venus positive or Venus negative without sample blinding.

### HFF transfection.

HFFs were seeded at a density of 7.5 × 10^4^ cells per well on a 13-mm glass coverslip 24 h before transfection with Lipofectamine 2000 (Thermo Fisher). For each well, 1.5 μl Lipofectamine 2000 was mixed with 800 ng plasmid DNA in 100 μl Opti-MEM (Thermo Fisher). Cells were fixed in 3% FA at 18 h posttransfection.

### Immunofluorescence analysis.

For immunofluorescence analysis, cells were quenched in 50 mM ammonium chloride for 15 min. For transfection experiments and initial RHΔ*ku80*+SFP1::myc or RHΔ*ku80*+GRA29::HA infections, samples were permeabilized for 3 min in 0.1% Triton X-100 and blocked with 2% BSA in PBS. For subsequent infection experiments, samples were permeabilized and probed in a solution containing 0.05% saponin and 0.2% fish gelatin in PBS. Infected or transfected cells were probed with mouse anti-myc (1:1,000) (catalog no. 05-724; Millipore), rat anti-HA (1:1,000) (catalog no. 11867423001; Roche), mouse anti-Flag (1:1,000) (catalog no. F1804; Sigma), rabbit anti-GRA29 (1:4,000) (33), rabbit anti-SFP1 serum (1:1,000), mouse anti-SFP1 (1:200), or mouse anti-CST1 (1:100) (gift from Louis Weiss), followed by Alexa Fluor 488- or 564-conjugated secondary antibodies (1:2,000) (Life Technologies). Additionally, cells were labeled with 4′,6-diamidino-2-phenylindole (DAPI) (1:1,000 dilution of 5 mg/ml) (Sigma-Aldrich), Alexa Fluor 647-conjugated phalloidin (1:100) (catalog no. A22287; Invitrogen), or rhodamine-conjugated Dolichos biflorus agglutinin (1:200) (catalog no. RL-1302; Vector Labs). Antibodies/stains were diluted in 2% BSA–PBS, samples were incubated for 1 h, and coverslips were subsequently washed and mounted with ProLong Gold antifade mountant (catalog no. P36934; Thermo Fisher). Localizations were visualized on a Nikon Ti-E microscope or a Leica SP5 confocal microscope.

### Superresolution microscopy.

For superresolution microscopy, 24- by 24-mm no. 1.5H coverslips were washed in 1 M HCl overnight and rinsed in water before coating with 1% (wt/vol) porcine gelatin (catalog no. G1890; Sigma) in 6-well plates. Confluent HFF monolayers were infected at an MOI of 0.5 and centrifuged at 180 × *g* for 3 min to synchronize infection. Samples were fixed and processed as described above except that antibody dilutions were passed through 0.45-μm filters and an additional final fixation step (10 min, 2% FA) was included after secondary antibody incubation. Superresolution microscopy imaging was performed using an OMX v3 structured illumination microscope (Applied Precision/GE Healthcare) equipped with 405-, 488-, and 592.5-nm diode lasers; electron-multiplying charge-coupled-device (CCD) cameras (Cascade II 512 by 512; Photometrics); and a 100×/1.40-numerical-aperture (NA) PlanApochromat oil immersion lens objective (Olympus). Three-dimensional structured illumination microscopy (3D-SIM) image stacks were reconstructed and aligned using softWoRx (Applied Precision/GE Healthcare).

### Correlative light and electron microscopy.

HFFs were seeded on a 3.5-cm dish with a gridded coverslip (MatTek) and infected, fixed, and processed as described above. Regions of interest with GRA29-HA cage structures were selected and imaged by SIM. Tiled images of the surrounding area were generated by confocal imaging to aid in identifying the region by EM. The cells were then fixed and prepared for EM as described below. The light and electron microscopy overlay was generated by using the TurboReg plug-in of Fiji software, with an affine transformation using the parasite nuclei and apicoplasts as landmarks, and by selecting high-intensity pixels in Photoshop using the magic wand tool with tolerance set to 40.

### EM sample preparation and imaging.

Cells were fixed in a solution containing 2.5% glutaraldehyde and 4% formaldehyde in 0.1 M phosphate buffer (PB) at room temperature (RT) for 30 min, postfixed in 1% reduced osmium tetroxide for 1 h, stained with 1% tannic acid in 0.05 M PB for 45 min, and quenched with 1% sodium sulfate in 0.05 M PB for 5 min. For CLEM experiments, the gridded coverslip was then removed from the MatTek dish. After washing in PB and water, the cells were dehydrated in 70, 90, and 100% ethanol (twice for 5 min each) before infiltration with a 1:1 mix of propylene oxide and Epon resin (Taab Embed 812) for 1 h and then twice for 90 min with Epon resin. Finally, coverslips were embedded in resin for 24 h at 60°C, sectioned (70 to 75 nm), and stained with lead citrate. Images were acquired using a Tecnai Spirit Biotwin transmission electron microscope (Thermo Fisher Scientific).

### *In vivo* infections.

C57BL/6 (wild-type) mice were bred and housed under pathogen-free conditions in the biological research facility at The Francis Crick Institute in accordance with the Home Office UK Animals (Scientific Procedures) Act 1986. All work was approved by the UK Home Office (project license PDE274B7D) and The Francis Crick Institute Ethical Review Panel and conformed to European Union directive 2010/63/EU. All mice used in this study were male and between 7 and 9 weeks old.

Mice were infected with PruΔ*ku80*, PruΔ*sfp1*, or PruΔ*gra29* tachyzoites by intraperitoneal (i.p.) injection with either 3E5 or 5E4 parasites in 200 μl PBS on day 0 to assess mouse survival. Mice were monitored and weighed regularly for the duration of the experiments.

### Mass spectrometry.

**(i) SFP1 transfection and immunoprecipitation.** A total of 2.8 × 10^6^ HFFs were seeded into a 15-cm dish 24 h before transfection with 44 μg pcDNA-SFP1 using Lipofectamine 3000 (Thermo Fisher) according to the manufacturer’s instructions. At 18 h posttransfection, cells were lysed on ice with 2 ml IP buffer (Thermo Scientific) for 30 min, passed through a 23-gauge needle, and clarified by centrifugation. The supernatant was incubated with 60 μl of polyclonal SFP1 antibody-coated Fastflow protein G-Sepharose (GE Healthcare). Sepharose bead complexes were washed in IP buffer followed by Tris-buffered saline, and proteins were eluted by boiling in Laemmli buffer.

**(ii) In-gel digestion and LC-MS/MS.** Eluted proteins were separated by SDS-PAGE until the running front had migrated approximately 2 cm into the gel (12% NuPAGE; Invitrogen) and stained with colloidal Coomassie (InstantBlue; Expedeon). After the excision of 8 horizontal gel slices per lane, proteins were in-gel digested with trypsin (Promega/Pierce) using a Janus liquid-handling system (PerkinElmer). Tryptic peptides were analyzed by liquid chromatography-mass spectrometry (LC-MS) using an Orbitrap Velos mass spectrometer coupled to an Ultimate 3000 ultrahigh-performance liquid chromatography (uHPLC) instrument equipped with an Easy-Spray nanosource (Thermo Fisher Scientific) and acquired in data-dependent mode.

**(iii) MS data processing and analysis.** The data were searched against Toxoplasma gondii and Homo sapiens (both UniProt) databases using the Andromeda search engine. Raw data were processed with MaxQuant (version 1.5.2.8). Cysteine carbamidomethylation was selected as a fixed modification. Methionine oxidation, acetylation of the protein N terminus, and phosphorylation (S, T, and Y) were selected as variable modifications. The enzyme specificity was set to trypsin with a maximum of 2 missed cleavages. The data sets were filtered on posterior error probability to achieve a 1% false-discovery rate (FDR) on the protein, peptide, and site levels. Data were further analyzed with Perseus (version 1.5.0.9). The data were filtered to remove common contaminants and identifications (IDs) originating from reverse decoy sequences and identified only by site. The log_10_ values for peptide intensities were then determined, and all SFP1 peptides (58% sequence coverage) were analyzed based on their modification state. No phosphorylated peptides were found.

### Tachyzoite versus bradyzoite phosphoproteomes.

**(i) Infection conditions.** Confluent HFF monolayers were grown in 15-cm dishes and infected with PruΔ*hxgprt*. For tachyzoite samples, three dishes were infected with 1.3 × 10^7^ parasites and incubated for 27 h. For bradyzoite samples, three plates were infected with 1.1 × 10^7^ parasites for 3.5 h before extensive washing and the addition of switch medium (described above). Bradyzoite infection samples were incubated as described above for 3 days, with fresh switch medium added daily.

**(ii) Cell lysis and protein digestion.** Lysis was performed in a solution containing ice-cold 8 M urea, 75 mM NaCl, and 50 mM Tris (pH 8.2) supplemented with protease (complete mini; Roche) and phosphatase (Phos Stop; Roche) inhibitors. Lysis was followed by sonication to reduce sample viscosity (30% duty cycle, with three 30-s bursts, on ice). The protein concentration was measured using a bicinchoninic acid (BCA) protein assay kit (Pierce). Lysates (1 mg each) were subsequently reduced with 5 mM DTT for 30 min at 56°C and alkylated in the dark with 14 mM iodoacetamide for 30 min at RT. Following iodoacetamide quenching with 5 mM DTT for 15 min in the dark, the lysates were diluted with 50 mM ammonium bicarbonate to <4 M urea and digested with LysC (Promega) for 2 to 3 h at 37°C. The lysates were further diluted with 50 mM ammonium bicarbonate to <2 M urea and digested with trypsin (Promega) overnight at 37°C. After digestion, samples were acidified with trifluoroacetic acid (TFA) (Thermo Fisher Scientific) to a final concentration of 1% (vol/vol). All insoluble material was removed by centrifugation, and the supernatant was desalted on Sep-Pak C_18_ cartridges (Waters).

**(iii) TMT labeling.** Samples were dissolved at 1 mg/ml in a solution containing 50 mM Na-HEPES (pH 8.5) and 30% (vol/vol) acetonitrile and labeled with the respective TMT reagents (Thermo Fisher Scientific) (2.4 mg reagent/1 mg sample) for 1 h at RT. Labeling was then quenched with 0.3% hydroxylamine for 15 min at RT, and samples were acidified (pH ∼2) with formic acid. After verification of labeling efficiency via mass spectrometry, the lysates were mixed in a 1:1 ratio, vacuum dried, and desalted on Sep-Pak C_18_ cartridges.

**(iv) Phosphopeptide enrichment.** Desalted and vacuum-dried samples were solubilized in 1 ml of loading buffer (80% acetonitrile, 5% TFA, 1 M glycolic acid) and mixed with 5 mg of TiO_2_ beads (Titansphere, 5 μm; GL Sciences Japan). Samples were incubated for 10 min with agitation, followed by a 1-min spin at 2,000 × *g* to pellet the beads. The supernatant was removed and used for a second round of enrichment as explained below. Beads were washed with 150 μl loading buffer, followed by two additional washes, the first with 150 μl 80% acetonitrile–1% TFA and the second with 150 μl 10% acetonitrile–0.2% TFA. After each wash, beads were pelleted by centrifugation (1 min at 2,000 × *g*), and the supernatant was discarded. Beads were dried in a vacuum centrifuge for 30 min, followed by two elution steps at high pH. For the first elution step, beads were mixed with 100 μl of 1% (vol/vol) ammonium hydroxide, and for the second elution step, beads were mixed with 100 μl of 5% (vol/vol) ammonium hydroxide. Each time, the beads were incubated for 10 min with agitation and pelleted at 2,000 × *g* for 1 min. The two eluates were removed following each spin and subsequently pooled before undergoing vacuum drying. The supernatant from the TiO_2_ enrichment was desalted on Sep-Pak C_18_ cartridges, and the High Select Fe-NTA phosphopeptide enrichment kit (Thermo Fisher Scientific) was used for a second round of enrichment according to the manufacturer’s instructions.

**(v) Sample fractionation and desalting.** Combined TiO_2_ and Fe-NTA phosphopeptide eluates (phosphoproteome) as well as 100 μg of the postenrichment supernatant (total proteome) were fractionated using the Pierce high-pH reversed-phase kit (Thermo Fisher Scientific) according to the manufacturer’s instructions. The resulting fractions, 8 for each phospho- and total proteome, were taken to dryness by vacuum centrifugation and further desalted on a stage tip using Empore C_18_ discs (3M). Briefly, each stage tip was packed with one C_18_ disc and conditioned with 100 μl of 100% methanol, followed by 200 μl of 1% TFA. The sample was loaded in 100 μl of 1% TFA, washed 3 times with 200 μl of 1% TFA, and eluted with 50 μl of 50% acetonitrile–5% TFA. The desalted phosphoproteome and total proteome fractions were vacuum dried in preparation for LC-tandem MS (MS/MS) analysis.

**(vi) LC-MS/MS and data processing.** Samples were resuspended in 0.1% TFA and loaded onto a 50-cm Easy-Spray PepMap column (75-μm inner diameter, 2-μm particle size; Thermo Fisher Scientific) equipped with an integrated electrospray emitter. Reverse-phase chromatography was performed using the RSLC nano U3000 instrument (Thermo Fisher Scientific) with a binary buffer system (solvent A, 0.1% formic acid and 5% DMSO; solvent B, 80% acetonitrile, 0.1% formic acid, and 5% DMSO) at a flow rate of 250 nl/min. The samples were run on a linear gradient of 5 to 60% solvent B in 150 min, with a total run time of 180 min, including column conditioning. The nanoscale LC (nanoLC) instrument was coupled to an Orbitrap Fusion Lumos mass spectrometer using an Easy-Spray nanosource (Thermo Fisher Scientific). The Orbitrap Fusion Lumos instrument was operated in data-dependent mode using 3 acquisition methods. For the phospho MS2 method, high-energy collisional dissociation (HCD) MS/MS scans (*R* = 50,000) were acquired after an MS1 survey scan (*R* = 120,000) using an MS1 target of 4E5 ions and an MS2 target of 2E5 ions. The number of precursor ions selected for fragmentation was determined by the “Top Speed” acquisition algorithm with a cycle time of 3 s and dynamic exclusion of 60 s. The maximum ion injection time utilized for MS2 scans was 86 ms, and the HCD collision energy was set at 38. For the phospho MS3 method, collision-induced dissociation (CID) MS/MS scans (*R* = 30,000) were acquired after an MS1 survey scan with the same parameters as the ones described above. The MS2 ion target was set at 5E4, with multistage activation of neutral loss (H_3_PO_4_) enabled. The maximum ion injection time utilized for MS2 scans was 80 ms, and the CID collision energy was set at 35. The HCD MS3 scan (*R* = 60,000) was performed with synchronous precursor selection enabled to include up to 5 MS2 fragment ions. The ion target was 1E5, the maximum ion injection time was 105 ms, and the HCD collision energy was set at 65. The proteome MS2 method was the same as the one for phospho MS2 with the following modification to MS2 target 5E4 ions. Acquired raw data files were processed with MaxQuant (47) (version 1.5.2.8), and peptides were identified from the MS/MS spectra searched against Toxoplasma gondii (ToxoDB) and Homo sapiens (UniProt) proteomes using the Andromeda (48) search engine. TMT-based experiments in MaxQuant were performed using the “reporter ion MS2 or MS3” built-in quantification algorithm, with the reporter mass tolerance set to 0.003 Da. Cysteine carbamidomethylation was selected as a fixed modification. Methionine oxidation, acetylation of the protein N terminus, deamidation (N, Q), and phosphorylation (S, T, and Y) were selected as variable modifications. The enzyme specificity was set to trypsin with a maximum of 2 missed cleavages. The precursor mass tolerance was set to 20 ppm for the first search (used for mass recalibration) and to 4.5 ppm for the main search. The “Match between runs” option was enabled for fractionated samples (time window of 0.7 min). The data sets were filtered on posterior error probability to achieve a 1% false-discovery rate on the protein, peptide, and site levels. Data were further analyzed as described in Results and in Data Set S1 using Microsoft Office Excel 2016 and Perseus (49) (version 1.5.0.9).

### Data availability.

The data behind the manuscript are freely available. The processed proteome and phosphoproteome data are included in Data Set S1, and the raw mass spectrometry data have been deposited to the ProteomeXchange Consortium via the PRIDE (50) partner repository with the data set identifier PXD019729.
