# Supplementary figures and images for: Phosphorylation of Toxoplasma gondii Secreted Proteins during Acute and Chronic Stages of Infection
Source: mSphere. 2020 Sep 9;5(5):e00792-20. doi: 10.1128/mSphere.00792-20 (PMC7485689; doi:10.1128/mSphere.00792-20)

Figure S1

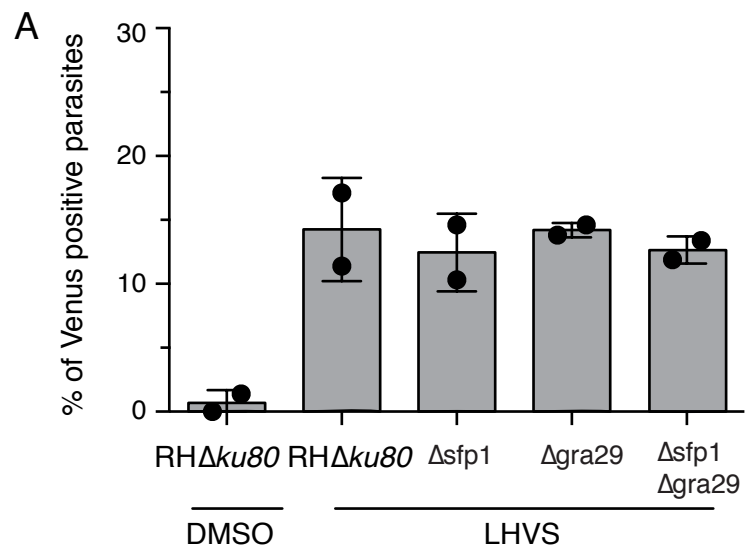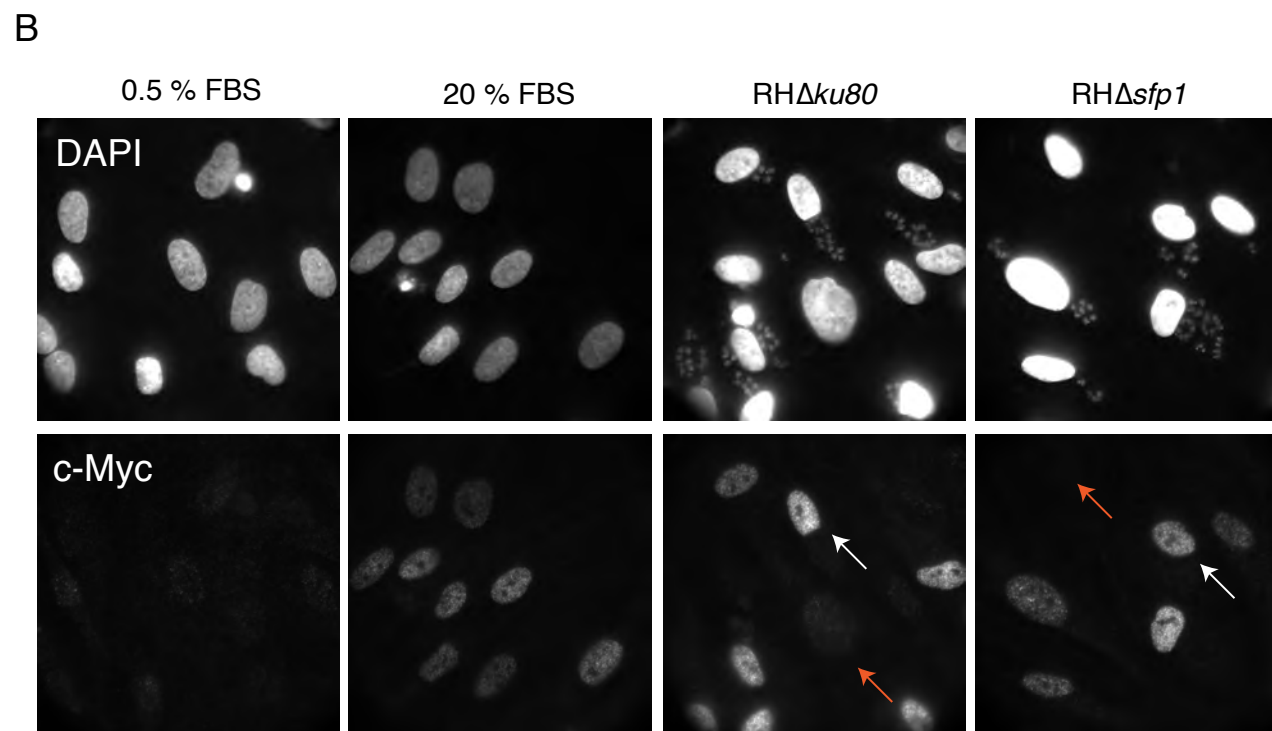

Supplement: FIG S1 [file mSphere.00792-20-sf001.pdf]

# Figure S3

A RH $\Delta ku80$  +GRA29-HA

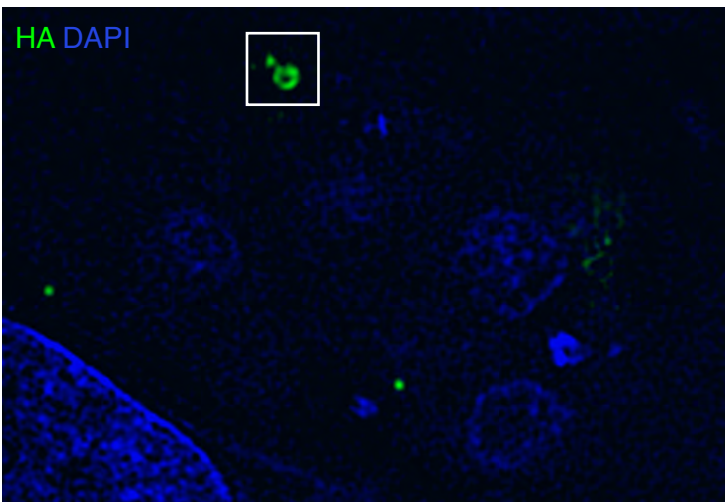

B

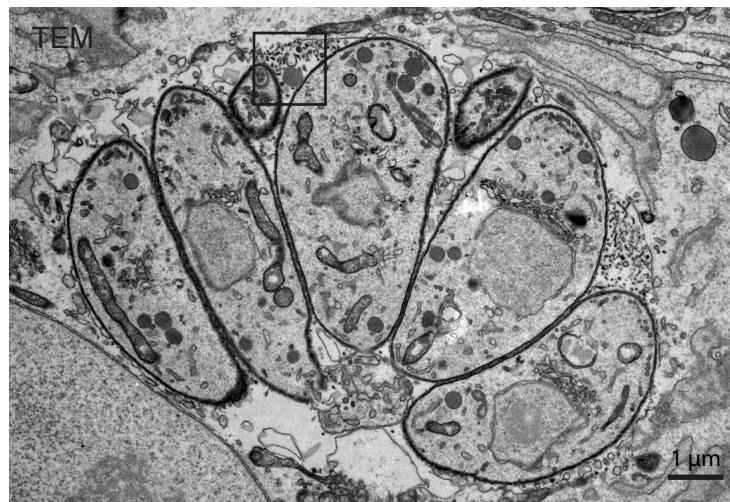

C

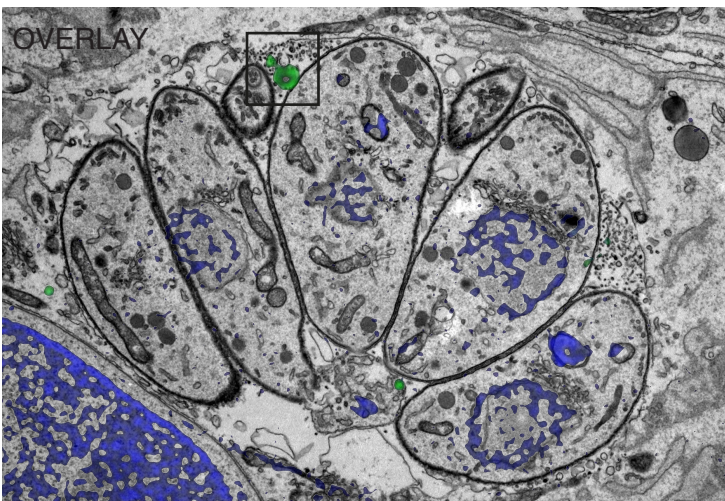

D

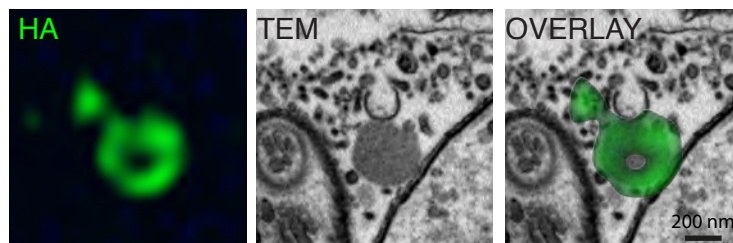

Supplement: FIG S3 [file mSphere.00792-20-sf003.pdf]

Figure S4

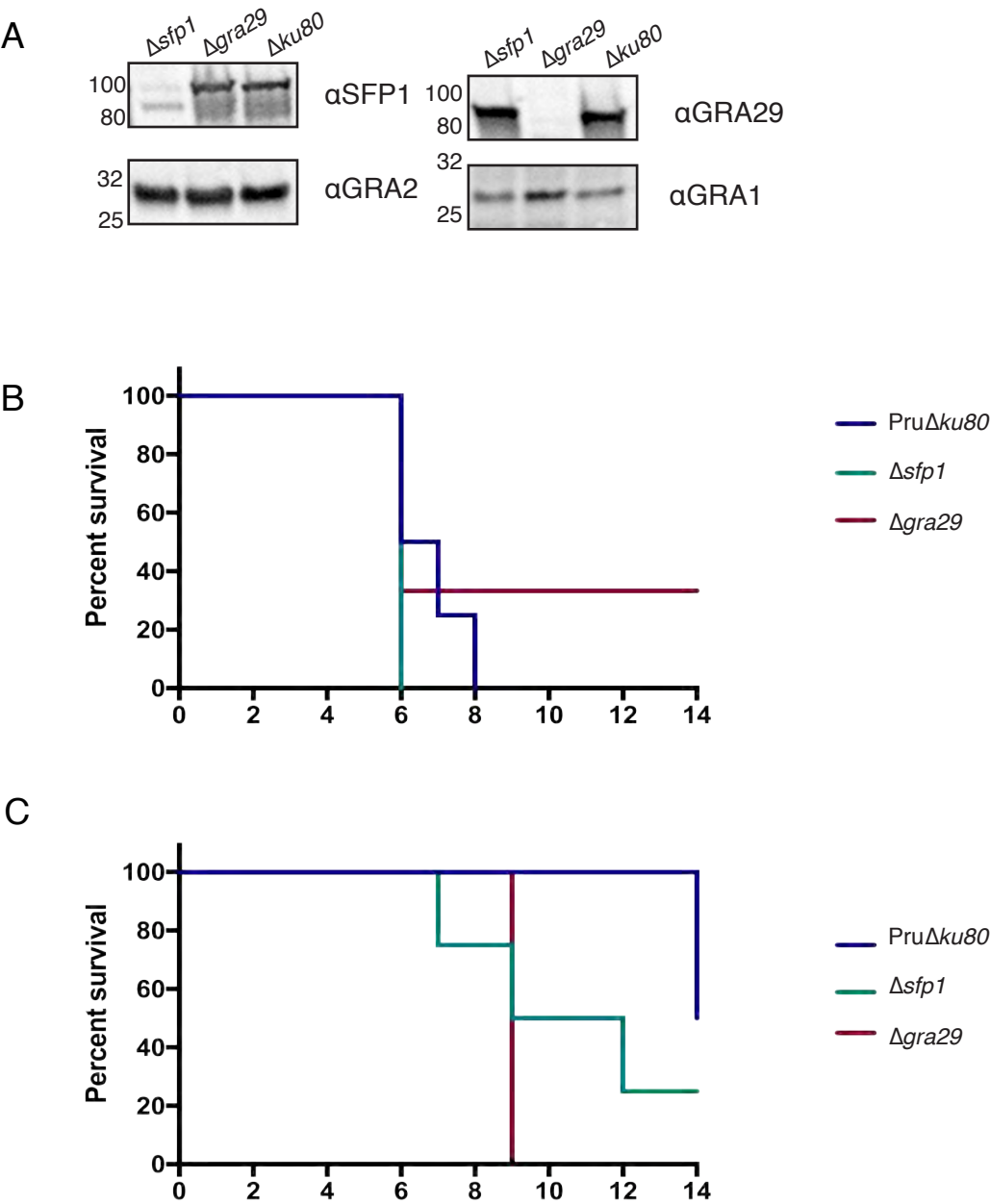

Supplement: FIG S4 [file mSphere.00792-20-sf004.pdf]

Figure S5

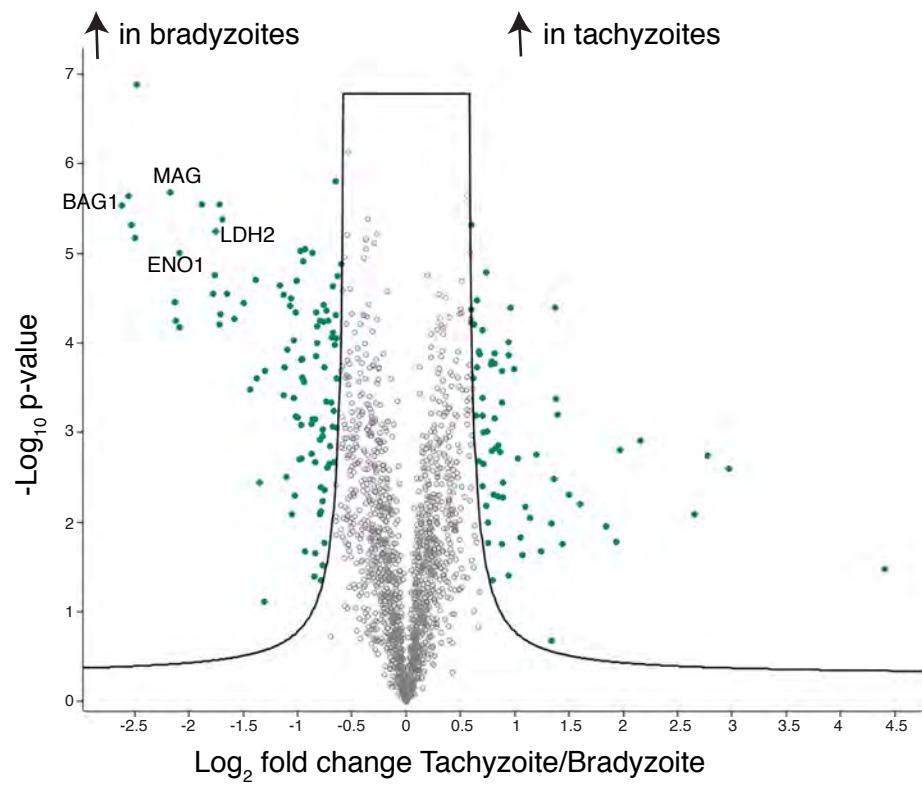

Supplement: FIG S5 [file mSphere.00792-20-sf005.pdf]
