# Supplementary material for: Phosphorylation of Toxoplasma gondii Secreted Proteins during Acute and Chronic Stages of Infection
Source: mSphere. 2020 Sep 9;5(5):e00792-20. doi: 10.1128/mSphere.00792-20 (PMC7485689; doi:10.1128/mSphere.00792-20)
Supplement: FIG S2 [file mSphere.00792-20-sf002.pdf]

Figure S2

```
SFP1      MSHKTMAKSGGRSTGAVRSHDAGSPSAPVASKSRPWRLGCMVVSLAAFAVAISTPSLSPA 60
GRA29     -------------------------------------MRPLRVTVALFWAMVAVVGFWAS 23
                                               :  : *::* * . ::. .: .:

SFP1      PASLASFSGLQPAEAAG--LVASMKRAANQGWRTGARWLCTVFGLQYEHVKSVFRRS-GR 117
GRA29     PDSRLTVRGPQFADAATRGVVKSLKRAMNQGWRNGAHSICAILGCDYEHVKAHLTHKPTF 83
          * *  :. * * *:**   :* *:*** *****.**: :*:::* :*****: : :.

SFP1      HLELETLSEEVDRYLQLVEAAGHNEGNLPDAYQERGVHLVLAAQELFSRVLGKRVELPAR 177
GRA29     FHSFKAVCAQLDEFEEEVTHAKMREEDLTPSQREYRWLLLSKGAALINDALGTQFEVPAS 143
          . .::::. ::*.: : *  *  .* :*. : :*    *:  .  *:. .**.:.*:**

SFP1      YKAAARRFEAMKKKKKDSGSRKGPAPESATEAPAPAEPTGSDVSSSGAEARPQAESPAKE 237
GRA29     YVPHNAKNRHYIPGQRGEGD---------------------------------------- 163
          * .   : .     ::..*.

SFP1      ASKPEAEAVPTAASSLVAVPMRDRAFEESANERLRALKARHEADVSAIVDEANTRLKQYQ 297
GRA29     -DKEKLKTRMRGLQVVVPLPTLEEIQKD-GMDRLAAVREKHMAQIKELEDARHRKFLLLQ 221
           .* : ::   . . :*.:*  :.  :: . :** *:: :* *::. : *  : ::   *

SFP1      EEHKMTYDKKISHLRAQQHAELDQALRELQEETLRFEDGKKQYEVVSALLTEAKAGIAVL 357
GRA29     EEHDRQTDQLVSELRLKHTEEMDTIRRELAEERRLFEQSVKVWDAVSRLLTDAHVGIETL 281
          ***.   *: :*.** ::  *:*   *** **   **:. * ::.** ***:*:.** .*

SFP1      SEGLQFIGANFYSIDQQIDKVAEEQKELEQADGDKAMIQKAFLVLQHAGLAQEQGAQAAI 417
GRA29     TDRVHTMSQHVFKLNVFYEKLHS----FQQTAKENPTGAAPYSALSYAFQSEAAVDQAMH 337
          :: :: :. :.:.::   :*: .    ::*:  ::.    .: .*.:*  ::    **

SFP1      AIRTRSAEIADVVAQLEELLSRADTALSTAPVG---SLFESQQQGYSEYLRSYQVNVAES 474
GRA29     QIVAIYQEASAGVARVQEILNQADGVMATAPTRDDAAWFQQRQRGYKDRLESFVNLLSDL 397
           * :   * :  **:::*:*.:** .::***.    : *:.:*:**.: *.*:   :::

SFP1      KEKVTSFEQEADAKLLALTYRLEQLQAKAVGNLGAVTEKLAAEYSMIQNKAQELEQGYQD 534
GRA29     SVRLEDYMQTAEERKLVTLFLGDEVRTQILALLQERTTEWSTRLATLRERTQGIETAANK 457
          . :: .: * *: : *.  :  :::::: :. *   * : ::. : :::::* :* . :.

SFP1      FFDACAHVEEQTFNTAKSAVEDRATFADLYSSLQAEFNNLQVTLERQGENVKQLNADVSN 594
GRA29     FFESSTQLDRTTGEVVSAVLQNPEAFLVQVSTYATESGTLRSAAGVIEEDLKAVVKALQD 517
          **::.::::. * :...:.:::  :*    *:  :* ..*: :     *::* :   :.:

SFP1      VEKIAAVFMEGLNSAPLSREALAQLNAQPVAEMTRFFSGYKQQVSVASTALHQRYQTTQD 654
GRA29     LGREMTGLTEGVASAQLPPAVLDFLGLPGLFNALQVDMQVKAKGQQLLQLIKGRLSQTGE 577
          : :  : : **: ** *.  .*  *.   : :  :.    * : .     :: * . * :

SFP1      LRRKLDLLHREIRVLADPATLERLEAEVMSLVADLEAKQRDQVEADAETKQARAVVQTLQ 714
GRA29     ITRKLEMLKSEVKNIRSPAVREQAEWALTEASTKLQDVLRTKAEVELEIQQINLQLQQVQ 637
          : ***::*: *:: : .**. *: *  : .  :.*:   * :.*.: * :* .  :* :*

SFP1      KDLRQLEQRLMTERRLAGRAGLAGAGASAAGG---SLFGGIGGTSASFAGSTLGSGFGAA 771
GRA29     GTVTQLDQRIAHERVVAQRAAAGGSAAAGLRGGSLALGGSATGSMVGLGGSVSGLGTDSR 697
            : **:**:  ** :* **. .*:.*:.  *   :* *.  *: ..:.**. * * .:

SFP1      NETVRELETQIRQLKARLQDAERELQNVQRRLTLVEKAVRDLQRDIDAKRRIIVEEQQKL 831
GRA29     -AAIADLEQKRQNEKEKIQELRHNLSSKQRESVSLDNEARRLEVEVANKQRELAEEQQRV 756
            :: :** : :: * ::*: .::*.. **. . ::: .* *: ::  *:* :.****::

SFP1      DRLQQLAAAVMGGPGSGVTGTLAAGLRGSVASGFRGSMASGLFPAGTIAAGLRGASVAGS 891
GRA29     LRVATLAQALTTGASPSIGMSMASMRGGLGLTGGLAGSSAALMTQGIMGSRAAGLLGGST 816
           *:  ** *:  *....:  ::*:   *   :*  .. ::.*:. * :.:   *   ..:

SFP1      LGGVGSRLGGFAGASMGRGLGSRAGGFG--ASGASKGPIPKPFTGDKN- 937
GRA29     AGLAGASMGGLAGGSMGGLGGAGMGGYGGGSMGGLGGRSTDGQLGRGTR 865
           * .*: :**:**.***   *:  **:*  : *.  *  ..   *  .
```
