# Supplementary material for: Phosphorylation of Toxoplasma gondii Secreted Proteins during Acute and Chronic Stages of Infection
Source: mSphere. 2020 Sep 9;5(5):e00792-20. doi: 10.1128/mSphere.00792-20 (PMC7485689; doi:10.1128/mSphere.00792-20)
Supplement: TABLE S1 [file mSphere.00792-20-st001.docx]

| Table S1. Oligonucleotides | | |  |
| --- | --- | --- | --- |
| Number | Description | Sequence | |
| 1 | pGRA SFP1 prom F | ttccgcgggcgggtttgaatgcaaggtttcgtgctgATCAAGCTTCTCCGGCAAGGCAGCGTGGAC | |
| 2 | pGRA SFP1 R | GCGGCTTTCCAGCCTGCGCATCACTTTCGTCGTAGTCTTAATTAAtcagttcttgtcccccgtgaagg | |
| 3 | pGRA-SFP1 myc F | GAACAGAAGCTCATCTCAGAAGAGGATCTGtgaTTAATTAAGACTACGACGAAAGTG | |
| 4 | SFP1 R (myc insertion) | gttcttgtcTcccgtgaagg | |
| 5 | SFP1_862 R | AGCCACGGAGCCTCGCAACCCTG | |
| 6 | pSFP1 Apa insert F | gctgcggtcatgggcGGgCCcggaagcggcgtgacc | |
| 7 | pSFP1 Apa insert R | ggtcacgccgcttccgGGcCCgcccatgaccgcagc | |
| 8 | pGRA GRA29 prom F | GCGGGTTTGAATGCAAGGTTTCGTGCTGATCaagcttCCGCCCagatctttcgtcggag | |
| 9 | pGRA GRA29-HA R | cgtagtccgggacgtcgtacgggtaccatggACGTGTCCCTCTTCCCAACTGTC | |
| 10 | pNTAP SFP1 54 F | cctggactacgacatccctaccaccgccagcgaattcACGCCCTCCCTGTCTCCCGCTC | |
| 11 | pNTAP SFP1 R | agAATAGGGCCCTCTAGATGCATGCTCGAgcggccgcTCAGTTCTTGTCCCCCGTGAAGG | |
| 12 | pNTAP SFP1 862 R | GCATGCTCGAGCGGCCGCTCAAGCCACGGAGCCTCGCAAC | |
| 13 | pNTAP GRA29 26 F | cgccctggactacgacatccctaccaccgccagcgaattcAGCCGCTTGACCGTGC | |
| 14 | pNTAP GRA29 R | TATAGAATAGGGCCCTCTAGATGCATGCTCGAGCGGCCGCTTAACGTGTCCCTCTTCCCAAC | |
| 15 | pNTAP GRA29 HA insert F | tacccgtacgacgtcccggactacgcggaattcAGCCGCTTGACCGTGCG | |
| 16 | pNTAP GRA29 HA insert R | gtcCATggtggatcactctagaAGCTTGGGTCTC | |
| 17 | pcDNA stop F | TGAgcggccgcTCGAGCATG | |
| 18 | GRA29 770 R | TGCACCAGTTGTTAGCGC | |
| 19 | pSAG1::cas9UG R | AACTTGACATCCCCATTTAC | |
| 20 | SFP1 5' gRNA F | TCGCGTCGAAGTCGCGGCCGGTTTTAGAGCTAGAAATAGC | |
| 21 | SFP1 3' gRNA F | GATCGGCCCTTTACTGGCGCGTTTTAGAGCTAGAAATAGC | |
| 22 | GRA29 5' gRNA F | AGGGCAACGGTCACACGTAAGTTTTAGAGCTAGAAATAGC | |
| 23 | GRA29 3' gRNA F | GGACCAGTTGTTTACATGGAGTTTTAGAGCTAGAAATAGC | |
| 24 | pSAG1::cas9 gRNA KpnI F | GTAATACGACTCACTATAGGGCGAATTGGGTACCCAAGTAAGCAGAAGCACGCT | |
| 25 | pSAG1::cas9 gRNA XhoI R | GTAAAAGCTTATCGATACCGTCGACCTCGAGAATTAACCCTCACTAAAGG | |
| 26 | pET28a SFP1 54F | tcacagcagcggcctggtgccgcgcggcagccatatgACGCCCTCCCTGTCTCCCGCTC | |
| 27 | pET28a SFP1 R | GTGGTGGTGGTGGTGctcgagtgcggccgcaagcttTCAGTTCTTGTCCCCCGTGAAGG | |
