# Supplementary material for: Phosphorylation of Toxoplasma gondii Secreted Proteins during Acute and Chronic Stages of Infection
Source: mSphere. 2020 Sep 9;5(5):e00792-20. doi: 10.1128/mSphere.00792-20 (PMC7485689; doi:10.1128/mSphere.00792-20)
Supplement: TEXT S1 [file mSphere.00792-20-s0001.docx]

**Synthetic DNA fragments**

**Generating pSFP1 variants**

SFP1 myc Gblock

CTCGACAGACTTCAACAGCTCGCGGCTGCGGTCATGGGCggGccCGGAAGCGGaGTGACCGGtACGCTCGCGGCAGGaTTGCGAGGCTCCGTGGCTTCGGGCTTCAGGGGGTCGATGGCTTCCGGCCTTTTCCCTGCGGGGACGATCGCTGCTGGaCTCCGAGGtGCaTCGGTCGCCGGaAGCCTGGGtGGAGTCGGtAGCCGCCTCGGtGGaTTCGCtGGaGCGTCCATGGGCCGCGGATTGGGCTCCAGAGCCGGGGGCTTTGGGGCATCCGGCGCCAGTAAAGGGCCGATCCCCAAGCCCTTCACGGGGGACAAGAACGAACAGAAGCTCATCTCAGAAGAGGATCTGtgaTTAATTAAGACTACGACGAAAGTGATGCGCAGGCTGGAAAGCCGCTG

SFP1 ALA myc Gblock

CTCGACAGACTTCAACAGCTCGCGGCTGCGGTCATGGGCGGgCCcGGAAGCGGaGTGACCGGtACGCTCGCGGCAGGaTTGCGAGGCTCCGTGGCTgCGGGCTTCAGGGGGgCGATGGCTgCaGGCCTTTTCCCTGCGGGGACGATCGCTGCTGGaCTCCGAGGaGCagCGGTCGCCGGagcaCTGGGaGGAGTCGGtgcaCGCCTCGGtGGaTTCGCtGGaGCGgCCATGGGCCGCGGATTGGGCgCaAGAGCCGGGGGCTTTGGGGCATCCGGCGCCgcTAAAGGGCCGATCCCCAAGCCCTTCgCGGGGGACAAGAACGAACAGAAGCTCATCTCAGAAGAGGATCTGtgaTTAATTAAGACTACGACGAAAGTGATGCGCAGGCTGGAAAGCCGCTG

SFP1 GLU myc Gblock

CTCGACAGACTTCAACAGCTCGCGGCTGCGGTCATGGGCGGgCCcGGAAGCGGaGTGACCGGtACGCTCGCGGCAGGaTTGCGAGGCTCCGTGGCTgaGGGCTTCAGGGGGgaGATGGCTgaaGGCCTTTTCCCTGCGGGGACGATCGCTGCTGGaCTCCGAGGaGCCgaGGTCGCCGGagagCTGGGaGGAGTCGGtgaaCGCCTCGGtGGaTTCGCtGGaGCGgaaATGGGCCGCGGATTGGGCgagAGAGCCGGGGGCTTTGGGGCATCCGGCGCCgaaAAAGGGCCGATCCCCAAGCCCTTCgaGGGGGACAAGAACGAACAGAAGCTCATCTCAGAAGAGGATCTGtgaTTAATTAAGACTACGACGAAAGTGATGCGCAGGCTGGAAAGCCGCTG

**Generating SFP1 KO**

SFP1 triple STOP repair

CCACACTGTGGTCGTCATGTCCCACAAGACCATGGCGAAGAGCGGGGGGCGTAGTACCGGCGCGGTCCGGTCGCATGACGCCGGGAGTCCTTCAGCGCCGGtcgcgtcgaagtcgcggccgTGACTAGCTAACGTCTCGGTTGCATGGTCGTCTCGTTGGCGGCCTTCGCTGTCGCCATCTCC
